# SOX21 suppresses glioblastoma growth by repressing AP-1 activity

**DOI:** 10.1038/s41419-026-08442-5

**Published:** 2026-01-31

**Authors:** Eltjona Rrapaj, Juan Yuan, Idha Kurtsdotter, Vsevolod Misyurin, Guido Alessandro Baselli, Johan Holmberg, Oscar Persson, Maria Bergsland, Jonas Muhr

**Affiliations:** 1 Department of Cell and Molecular Biology, Karolinska Institutet, Stockholm, Sweden; 2 SciLifeLab, Department of Microbiology, Tumor and Cell Biology, Karolinska Institutet, Stockholm, Sweden; 3 Department of Molecular Biology, Umeå University, Umeå, Sweden; 4 Department of Neurosurgery, Karolinska University Hospital, and Department of Clinical Neuroscience, Karolinska Institutet, Stockholm, Sweden; 5 Present Address: Department of Neuroscience, Karolinska Institutet, Stockholm, Sweden

**Keywords:** CNS cancer, Cancer stem cells

## Abstract

Treatment-resistant glioblastoma stem and precursor cells (GPCs) drive glioblastoma (GBM) growth and recurrence. Thus, targeting the molecular machinery that sustains GPCs in an undifferentiated and self-renewing state is a promising therapeutic strategy. The transcription factor SOX21 effectively suppresses the tumorigenic capacity of GPCs, but the mechanism by which SOX21 impedes GPC features is unknown. By engineering patient-derived GPCs with a transgenic TetOn system we show that SOX21 expression induces an anti-tumorigenic transcriptional program, aligning with clinical data demonstrating a positive correlation between SOX21 levels and improved GBM patient survival. Induced SOX21 expression in GPCs within pre-established GBM reduces their capacity to sustain tumor growth and significantly extends the survival of the orthotopically transplanted mice. Mechanistically, SOX21 functions as a tumor suppressor by binding a large set of AP-1-targeted chromatin regions, leading to epigenetic repression of AP-1-activated genes. Consistently, the anti-tumorigenic activities of SOX21 are largely replicated by AP-1 inhibitors, which decrease GPC proliferation and survival, while overexpression of the AP-1 family member, c-JUN, counteracts these effects. Our findings identify SOX21 as a key regulator that prevents GPC malignancy by targeting and repressing an AP-1-driven, tumor-promoting gene expression program. These results highlight SOX21-regulated pathways as promising therapeutic targets for GBM.

## Introduction

GBM is the most prevalent and aggressive form of primary brain tumors [1]. It is characterized by uncontrolled growth, cellular and molecular heterogeneity, and a high propensity for recurrence. One major reason why conventional therapies unavoidably fail is explained by the presence of treatment-resistant GPCs [2]. These cells exhibit extensive self-renewing capacity, supporting rapid tumor growth and high cellular plasticity, which contributes to inter- and intra-tumoral diversification [2, 3]. Additionally, GPCs possess strong tumor-initiating potential [4], underscoring the need for novel therapeutic strategies that target these cells to curb GBM progression and recurrence.

Stem cells, both in normal and malignant contexts, are regulated by an interplay of transcription factors that either maintain cells in an undifferentiated, self-renewing state or facilitate cellular differentiation and cell-cycle exit. Several transcription factors that promote the growth of healthy stem cells, including members of the *SOX*, *POU*, *AP-1*, *OLIG2*, and *E2F* families, have also been implicated in GPC maintenance and tumorigenicity [5–11]. For instance, SOX2, a key regulator of stem cells in developing and adult tissues, is also critical for GPC propagation and their tumorigenic capacity [12, 13]. Similarly, AP-1 family members, which have ubiquitous roles in regulating cell proliferation and survival [14], have been shown to promote stem cell-like properties and aggressiveness of GPCs [8, 9].

In contrast, the transcription factor SOX21 exhibits the opposite activity and reduces neural stem cell proliferation and promotes their differentiation [15]. Notably, SOX21 expression is significantly lower in high-grade gliomas compared to low-grade tumors [16]. Consistent with this, forced expression of SOX21 in human GPCs diminishes their ability to form secondary tumors following orthotopic transplantation into mice [16, 17]. Conversely, genetic ablation of *Sox21* in mouse brain stem cells strongly increases their propensity to form GBM-like tumors in an H-RAS/AKT-driven glioma model [16]. These gain- and loss-of-function experiments underscore the capacity of SOX21 in preventing oncogenic transformation of brain stem cells and demonstrate that forced expression of SOX21 in GPCs before transplantation curbs their capacity to initiate tumor formation in mice. However, from a therapeutic perspective, it is critical to examine whether SOX21 induction in already established GBM can block GPC propagation and thereby inhibit further tumor growth. Additionally, the molecular mechanisms by which SOX21 counterbalances genetic pathways that preserve GPC properties and promote GBM progression remain largely unexplained.

In this study, we generated an inducible expression system in patient-derived GPCs to investigate the therapeutic potential of SOX21. We demonstrate that the induction of SOX21 expression in GPCs reduces self-renewal and increase cell death. In established GBM, induced SOX21 expression, but not SOX2 expression, counteracts further tumor growth and significantly improves the survival of the orthotopically transplanted mice. AP-1 proteins promote GPC self-renewal and survival. We now show that SOX21 and AP-1 proteins interact and that a major portion of SOX21 binding is directed to chromatin regions targeted by the AP-1 family member c-JUN, resulting in repression of AP-1 activated genes and as a result an inhibition of GPC proliferation and survival.

## Results

### SOX21 expression is confined to GPCs and correlates with improved patient survival

**Fig. 1:**
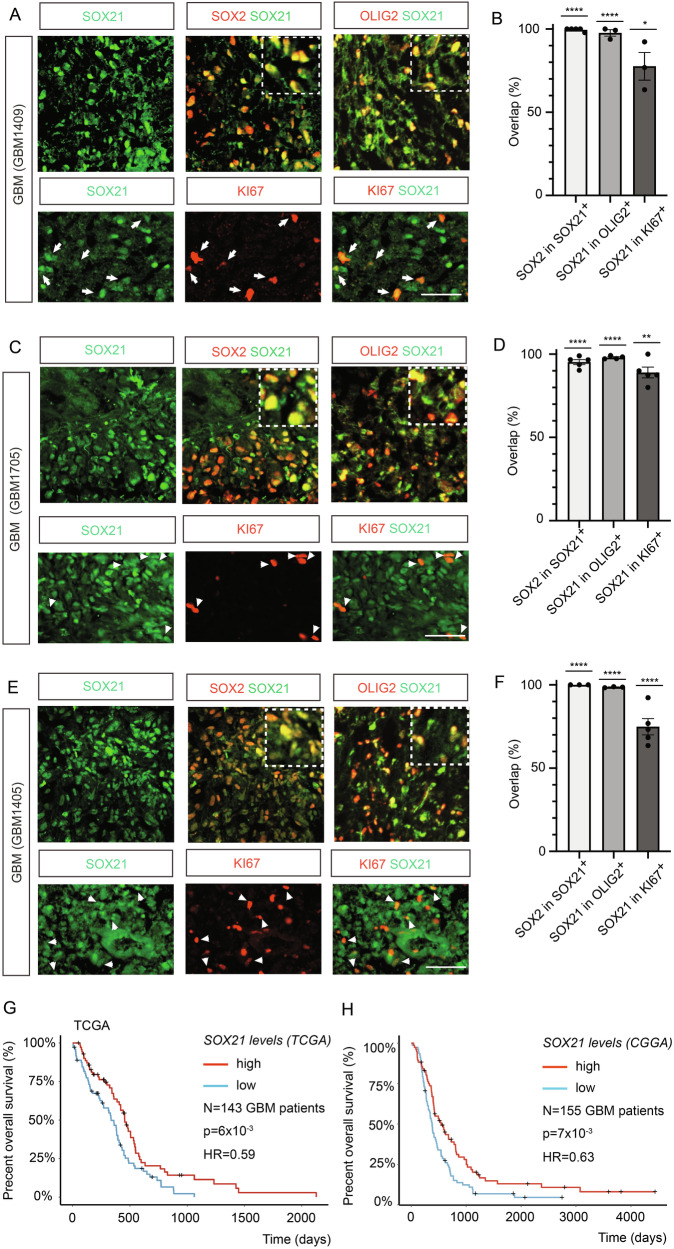
SOX21 expression is associated with improved survival in GBM patients. **A**–**F** Immunohistochemical and statistical analyses of GBM surgical specimens (GBM1409, GBM1705 and GBM1405) show that SOX21 protein (green) is predominantly expressed in SOX2^+^ cells (red) and is present in most OLIG2^+^ and KI67⁺ cells (red). Insets display magnified regions of the tumor. Scale bars: 50 µm in (**A**, **C**, **E**). Dots represent technical replicates (sections) analyzed per tumor. Kaplan-Meier survival analyses of IDH wildtype GBM patient cohorts from TCGA (*n* = 143) (**G**) and CGGA (*n* = 155) (**H**), classified according to the 2021 WHO (CNS5) criteria, demonstrate significantly improved overall survival in patients with high SOX21 expression (red line) compared with those with low expression (blue line). TCGA: *p* = 6 × 10⁻³, HR = 0.59; CGGA: *p* = 7 × 10⁻³, HR = 0.63.

To further explore the role of SOX21 in GBM, we examined its protein expression pattern in surgical GBM specimens (IDH wild type) using immunofluorescence. In three different tumors, SOX21 expression was largely restricted to SOX2^+^ cells (Fig. 1A–F). Additionally, SOX21 expression was detected in most of all cells expressing the stem and progenitor cell marker OLIG2 [11] and the proliferative marker KI67 (Fig. 1A–F). Hence, in human GBM the expression of SOX21 is confined to the GPC compartment.

Previous analyses of gene expression data sets from low-grade (WHO grade II and III) and high-grade (WHO grade IV, GBM) glioma have revealed an inverse correlation between SOX21 expression levels and glioma malignancy [16]. To examine if the expression levels of *SOX21* also hold a prognostic value for GBM patient survival, we divided the IDH wild type GBM data sets from the “The Cancer Genome Atlas” (TCGA, samples classified according to WHO CNS5) [18], or the “Chinese Glioma Genome Atlas” (CGGA) into two groups, based on *SOX21* mRNA levels, and correlated these to the patient survival. Notably, Kaplan-Meier survival analyses revealed that patients with high *SOX21* expression exhibited significantly better overall survival, compared to those with low *SOX21* expression (Fig. 1G, H).

### Elevated SOX21 levels suppress GPC proliferation and survival

**Fig. 2:**
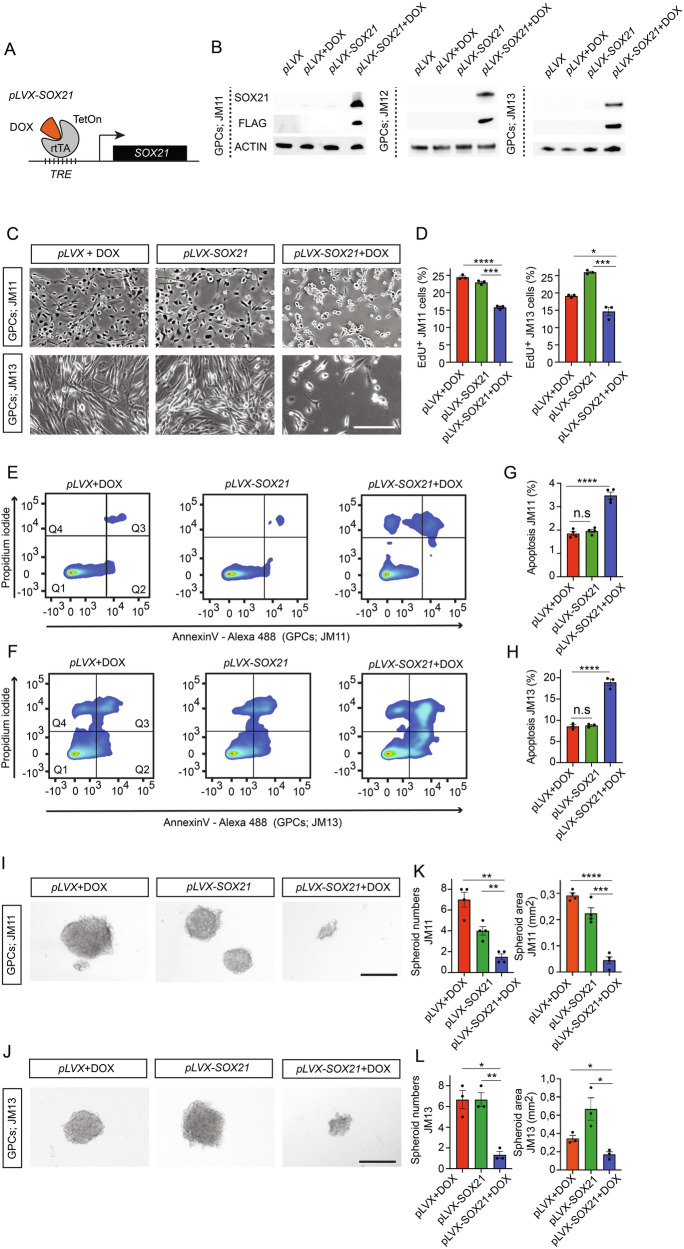
Effects of Inducible SOX21 Expression on GPC Growth. **A** Schematic representation of the tetracycline-inducible (Tet-On) SOX21 expression system. rtTA tetracycline-controlled transactivator, TRE tetracycline response element, DOX doxycycline. **B** Western blot analysis of FLAG-tagged SOX21 expression in GPC lines (JM11, JM12 and JM13) transduced with either control (*pLVX*) or inducible SOX21 expression vectors (*pLVX-SOX21*), cultured with or without DOX for 48 h. **C** Representative images showing reduced cell density in GPC monolayer cultures following DOX-induced SOX21 expression. Scale bar, 70 µm. **D** Quantification of EdU incorporation in control and SOX21-expressing GPCs (JM11 and JM13). Flow cytometry analysis of JM11 (**E**) and JM13 (**F**) GPCs transduced with control (*pLVX*) or SOX21-inducible (*pLVX-SOX21*) vectors and cultured with or without DOX for 4 days. Quadrant distributions: Q1 (live cells), Q2 (early apoptotic cells), Q3 (late apoptotic cells), and Q4 (necrotic cells). **G**, **H** Quantification of flow cytometry experiments showing increased apoptosis in GPCs following DOX-induced SOX21 expression, as assayed by Annexin V labeling. Representative images illustrating the sphere forming capacity of JM11 (**I**) and JM13 (**J**) GPCs with or without SOX21 induction. Scale bar, 500 µm. Bar graphs quantifying the number and size of spheroids formed by control or SOX21-induced JM11 (**K**) and JM13 (**L**) GPCs. Dots in bar graphs represent analyzed biological replicates.

The positive correlation between *SOX21* expression levels and patient survival suggests that high levels of SOX21 may exert an anti-tumorigenic function in high-grade gliomas. To address this possibility, we generated a tetracyclin-inducible (TetOn) lentiviral vector encoding a FLAG-tagged version of *SOX21* (*pLVX-SOX21*) and, as a control, a version lacking insert (*pLVX*) (Fig. 2A). The resulting viruses were stably transduced into primary GPCs derived from three different GBM tumors (Supplementary Table 1) GPCs hereafter referred to as JM11, JM12, and JM13. In comparison to 539 TCGA glioma samples, RNA-seq and UMAP (Uniform Manifold Approximation and Projection) analyses revealed that these GPCs exist in astrocyte-like (JM11), oligodendrocyte-progenitor-like (JM12), and mesenchymal-like (JM13) states, respectively (Supplementary Fig. 1). Following selection for successfully transduced cells, the expanded GPCs were confirmed, with immunoblotting and immunofluorescence, to specifically upregulate SOX21 upon doxycycline (DOX) treatment (Figs. 2B and 2A).

Focusing on JM11 and JM13, DOX-induced SOX21 expression, “*pLVX-SOX21* + DOX”, significantly reduced GPC density in monolayer cultures compared to controls, “*pLVX* + DOX” and “*pLVX-SOX21”* (Fig. 2C). Additionally, the incorporation of the thymidine analogue EdU revealed a marked decrease in the proportion of GPCs undergoing active proliferation upon SOX21 induction (Fig. 2D). Flow cytometry (FACS) based analysis further demonstrated that SOX21 upregulation significantly increased the proportion of early and late apoptotic GPCs, as evidenced by Annexin V expression and propidium iodide labeling (Fig. 2E–H). Consistently, elevated cytotoxicity levels in JM11 and JM13 GPCs after SOX21 induction (Supplementary Fig. 3A, B) were accompanied by increased expression of the pro-apoptotic markers BIM, BAX, BAK, as well as of the definitive apoptotic marker, cleaved CASPASE-3 [19] (Supplementary Fig. 3C, D). SOX21 induction also led to weak upregulation of the key ferroptosis marker GPX4 [20] in JM13, but not JM11 GPCs (Supplementary Fig. 3E, F).

To assess the impact of SOX21 expression on GPC clonal expansion capabilities, we performed sphere-forming assays. Within 10-12 days, DOX-induced SOX21 expression resulted in a notable reduction in both the number and size of the tumor spheres, compared to control conditions (Fig. 2I–L). Thus, increased levels of SOX21 suppress GPC proliferation, enhance apoptosis, and limit clonal expansion potential, underpinning its potential as a tumor suppressor in GPCs. Moreover, in support of its potential as a tumor suppressor SOX21 induction increased the sensitivity of both JM11 and JM13 GPCs to Temozolomide (TMZ), which is a primary chemotherapeutic drug used to treat GBM [21] (Supplementary Fig. 3G).

### Induced SOX21 expression in established GBM suppresses tumor progression

**Fig. 3:**
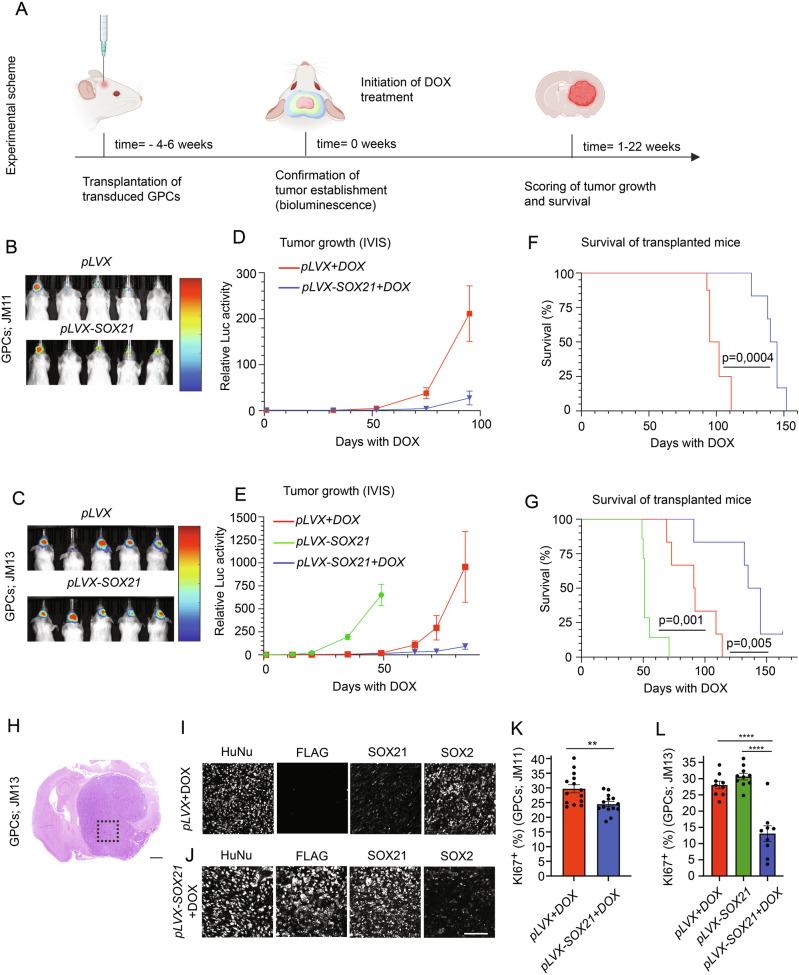
Induced SOX21 expression suppresses growth of pre-established GBM in mice. **A** Experimental timeline illustrating induction of SOX21 in pre-established tumors and subsequent assessment of tumor growth and animal survival. Bioluminescence imaging confirms successful tumor establishment prior to DOX administration in mice transplanted with control or SOX21-inducible JM11 (**B**) or JM13 (**C**) GPCs. Tumor growth curves show luciferase-based quantification of tumor burden in mice transplanted with control (red) or SOX21-inducible (green and blue) JM11 (**D**) or JM13 (**E**) GPCs after receiving control or DOX-supplemented food. Kaplan-Meier analyses show the survival of mice transplanted with control (red) or SOX21-inducible (green and blue) JM11 (**F**) or JM13 (**G**) GPCs after receiving control or DOX-supplemented food. **H** Representative H&E-stained section of an end-stage tumor from a DOX-treated mouse transplanted with control GPCs (JM13). Scale bar, 1,5 mm. **I**, **J** High-magnification immunohistochemical images showing expression of human nuclei (HuNu), FLAG-tagged SOX21, endogenous SOX21, and SOX2 in control or SOX21-induced JM13-derived tumors. Scale bar, 75 µm. Quantifications of KI67^+^ cells in JM11-derived (**K**) and JM13-derived (**L**) tumors demonstrate a reduction in proliferative activity upon SOX21 induction. Dots in bar graphs represent analyzed biological replicates.

**Fig. 4:**
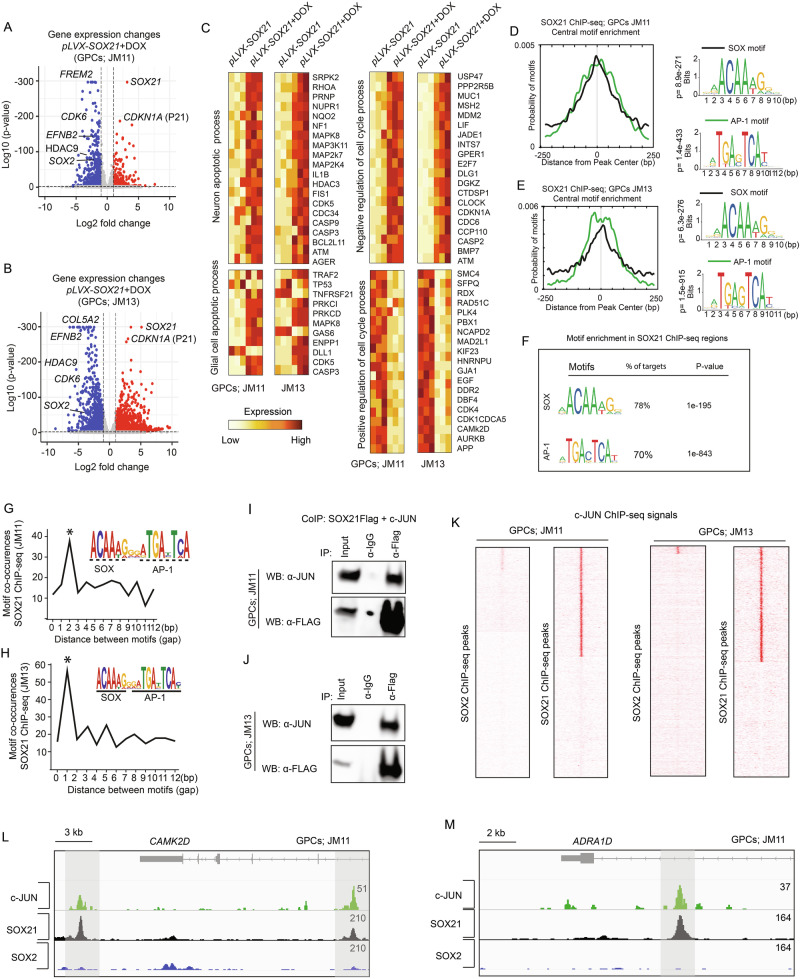
Transcriptomic impact of SOX21 expression in GPCs. Volcano plots displaying differential gene expression induced by SOX21 in JM11 (**A**) and JM13 (**B**) GPCs after 48 hours. Genes significantly upregulated (red) and downregulated (blue) are shown (false discovery rate < 0.01). **C** Heatmaps show gene sets and associated Gene Ontology (GO) terms deregulated upon SOX21 induction. **D**, **E** Enrichment analysis of SOX motifs (black line) and AP-1 motifs (green line) within SOX21 ChIP-seq peaks detected in JM11 (D) and JM13 (E) GPCs, with motif distances to the center of SOX21 peaks measured in base pairs (bp). P-values of best-matching SOX and AP-1 motifs are shown. **F** Enrichment of transcription factor binding motifs across SOX21 ChIP-seq regions in comparison with non-targeted control chromatin regions. Graphs illustrating the most significant spacing distances between centrally enriched SOX and AP-1 motifs in SOX21 ChIP-seq peaks detected in JM11 (**G**) and JM13 (**H**) GPCs. The gap between motifs is based on the spacing between the last nucleotide in the SOX motif and the first nucleotide in AP-1. **I**, **J** Co-immunoprecipitation assays showing interactions between FLAG-tagged SOX21 and c-JUN in JM11 and JM13 GPCs treated with DNase I. **K** Genomic mapping of c-JUN ChIP-seq reads within SOX21 ChIP-seq peak regions detected in JM11 and JM13 GPCs. **L**, **M** Genomic regions surrounding *CAMK2D* (**L**) and *ADRA1D* (**M**) genes, illustrating ChIP-seq binding profiles for c-JUN, SOX21, and SOX2 in JM11 GPCs.

To further evaluate the anti-tumorigenic effects of SOX21, we investigated its capability to restrict GBM growth in vivo. Previous studies have demonstrated that viral-mediated SOX21 expression in GPCs and glioma cell lines inhibits their tumor-initiating capacity in orthotopic mouse models [16, 17]. However, from a therapeutic perspective, it is critical to determine whether SOX21 induction in established GBM can block GPCs to promote further tumor growth. To address this issue, we transplanted JM11 and JM13 GPCs—engineered with either DOX-inducible SOX21 expression constructs or control vectors, along with a constitutive lentiviral Luciferase (Luc) reporter—into the striatum of NOD-SCID mice (Fig. 3A). Serving as an additional control, we also transplanted mice with Luc-expressing JM11 and JM12 GPCs carrying either a DOX-inducible SOX2 expression system (*pLVX-SOX2*; Supplementary Fig. 2B) or control vectors. Following tumor establishment, confirmed via luciferase activity measurements using an in vivo imaging system (IVIS) (Figs. 3A–C and 4A), mice were provided with DOX-supplemented food to induce transgenic expression [22].

Bioluminescence imaging every two weeks revealed that DOX-induced SOX21 expression induction (*pLVX-SOX21* + DOX) in either transplanted JM11 or JM13 GPCs significantly reduced GBM progression compared to controls (*pLVX* + DOX and *pLVX-SOX21*) (Fig. 3D, E). The suppression of tumor growth was further reflected by the significantly prolonged mean survival time of the transplanted mice upon SOX21 induction relative to controls (Fig. 3F, G). Immunohistochemical analysis of end-stage tumors confirmed retained transgene expression, although the proportion of transgene-expressing cells varied between tumors (Fig. 3H–J). Moreover, the DOX-induced SOX21 expression significantly decreased the proportion of KI67^+^ proliferative cells and SOX2^+^ GPCs (Fig. 3I–L). These findings demonstrate that SOX21 expression in pre-established GBM suppresses tumor progression by depleting proliferative GPCs. In contrast, DOX-induced expression of SOX2 in JM11 or JM12 GPCs had no significant effect on tumor growth or mice’s mean survival outcome (Supplementary Fig. 4A–F).

### SOX21 represses tumor-promoting genes and shares chromatin targets with AP-1

**Fig. 5:**
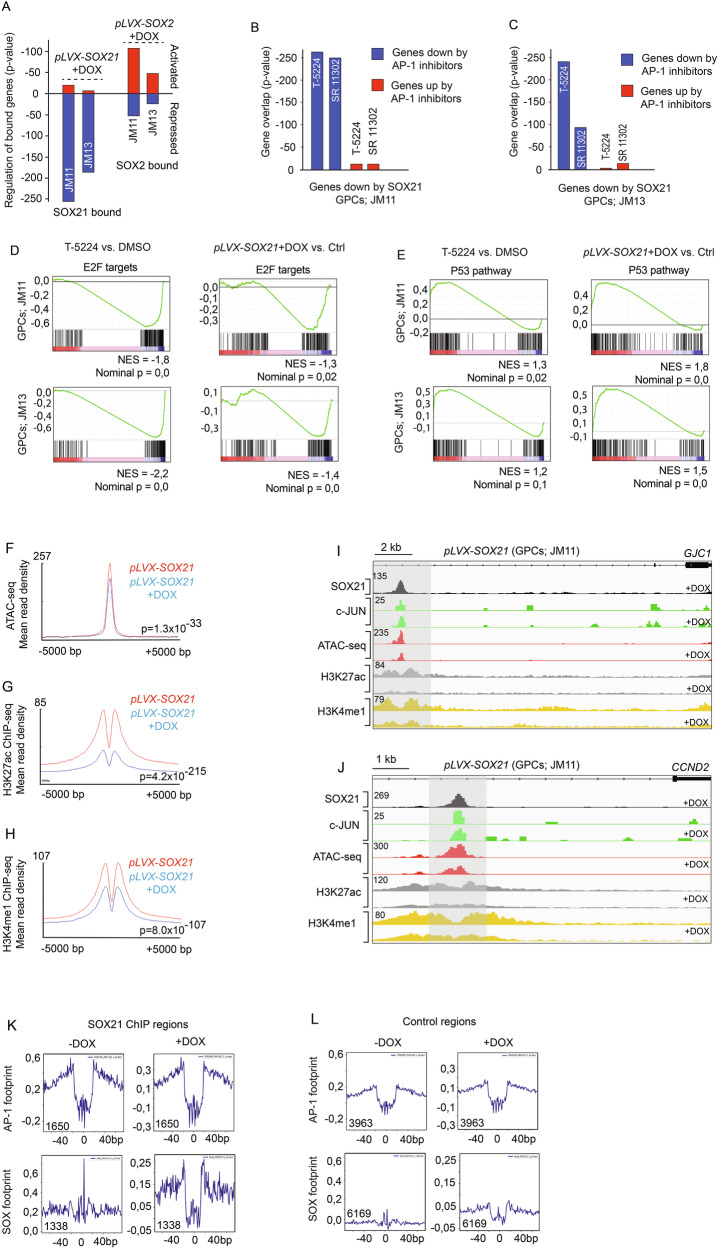
SOX21 mediated epigenetic repression of AP-1 targeted genes. **A** Bar graph showing the proportion of transcriptionally activated (red) and repressed (blue) SOX21- and SOX2-bound genes after DOX-induced SOX21 or SOX2 expression in JM11 and JM13 GPCs. p-values are based on overlap between regulated and bound genes. RNA-seq analyses of JM11 (**B**) and JM13 (**C**) GPCs reveal a significant overlap between genes downregulated by SOX21 and AP-1 inhibitors (T-5224 and SR 11302) (blue), but not among genes upregulated by AP-1 inhibitors (red). Gene Set Enrichment Analysis (GSEA) comparing transcriptional effects of AP-1 inhibition (T-5224) and SOX21 expression on E2F target genes (**D**) and the p53 pathway (**E**) in JM11 and JM13 GPCs (NES, Normalized Enrichment Score). Read mean density profiles for ATAC-seq (**F**), H3K27ac ChIP-seq (**G**), and H3K4me1 ChIP-seq (**H**) mapped onto SOX21 and c-JUN bound chromatin regions in control (red line) and SOX21-induced (blue line) JM11 GPCs. Distances from SOX21 ChIP-seq peak centers are indicated in base pairs (bp). **I**, **J** Representative genome browser tracks displaying SOX21 ChIP-seq (black), c-JUN ChIP-seq (green), ATAC-seq (red), H3K27ac ChIP-seq (gray), and H3K4me1 ChIP-seq (yellow) signal intensities in control and SOX21-induced JM11 GPCs. Numbers denote peak heights from the respective ATAC-seq and ChIP-seq datasets. **K**, **L** Footprint analyses of ATAC-seq data at AP-1 and SOX motifs, present within SOX21 ChIP-seq regions or control chromatin. Mean aggregate ATAC-seq signals are shown on y-axes and the number of analyzed motifs is stated in the graphs.

To elucidate the molecular mechanisms underlying SOX21-mediated tumor suppression, we profiled its genome-wide binding and transcriptional regulatory effects using RNA-seq and ChIP-seq in JM11 and JM13 GPCs, with SOX2 serving as a control. SOX21 induction for 48 hours resulted in the deregulation of over a thousand genes (Figs. 4A, B and 5A, B; Supplementary Tables 2, 3). Among the most significantly upregulated genes was *CDKN1A*, which encodes the tumor suppressor p21 (Figs. 4A and B and 5A–C). Gene ontology (GO) analysis revealed that SOX21 enhances the expression of apoptosis-related pathways, including “*neuron apoptotic process”*, “*glial cell apoptotic process*”, but also pathways such as “*negative regulation of cell cycle process*” (Figs. 4C and 5D). Conversely, SOX21 downregulated genes involved in glioma progression, such as *CDK6*, *EFNB2*, *HDAC9*, and *SOX2* (Figs. 4A and B and 5A, B) [23–27], and genes associated with “*Positive regulation of cell cycle process”*, which further supports its role as a tumor suppressor (Figs. 4C and 5D). SOX2 overexpression deregulated genes distinct from those altered by SOX21 (Supplementary Fig. 5E–G), although high levels of SOX2 downregulated the cell cycle gene *CCND1* [28]. Together, these findings highlight SOX21 as a transcriptional regulator that opposes tumor-promoting gene expression in GPCs.

**Fig. 6:**
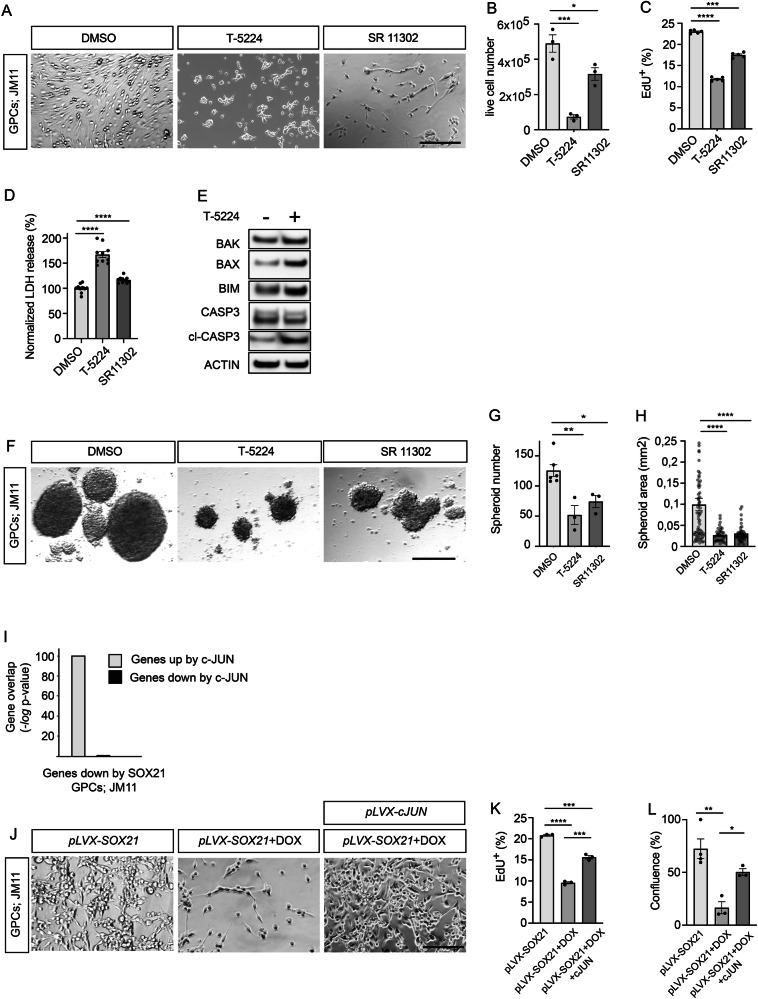
c-JUN expression counteracts SOX21-mediated suppression of GPCs features. **A** Representative images of JM11 GPCs treated for 4 days with DMSO (control), T-5224, or SR 11302. Scale bar, 50 µm. Quantification of cell viability (**B**), proliferation (EdU incorporation) (**C**) and cytotoxicity (LDH release) (**D**) following AP-1 inhibition. **E** Western blot analysis showing activation of apoptotic markers in JM11 GPCs cultured with the AP-1 inhibitor (T-5224) for 48 h. **F** Sphere formation assay of JM11 GPCs cultured with AP-1 inhibitors (T-5224 and SR 11302) or DMSO. Scale bar, 50 µm. Quantification of sphere numbers (**G**) and sphere area (**H**) in response to AP-1 inhibition. **I** RNA-seq analysis of JM11 GPCs showing strong enrichment of SOX21 downregulated genes among those upregulated by c-JUN (gray), but not among c-JUN downregulated genes (black). **J** Representative images show that enforced c-JUN expression in JM11 GPCs for 4 days rescues the reduction in cell number caused by DOX-induced SOX21 expression. Scale bar, 50 μm. Quantification of proliferation (EdU incorporation) (**K**) and viability (plate confluence) (**L**) following DOX-induced SOX21 expression either alone or in combination with forced c-JUN expression. Dots in bar graphs represent analyzed biological replicates or analyzed spheres.

ChIP-seq experiments revealed thousands of SOX21 and SOX2 binding sites (peaks), with high reproducibility across replicates (Supplementary Fig. 6A, B and Supplementary Table 4). Notably, approximately one-third of SOX21 binding sites were consistent across both JM11 and JM13 GPC lines, whereas SOX21 and SOX2 shared fewer than 10% of their binding sites (Supplementary Fig. 6C–E). Interestingly, while centrally located SOX binding motifs were present in both SOX21 and SOX2 peak regions (Figs. 4D, E and 6F, G), motif analysis also revealed a strong centrally enrichment of AP-1 transcription factor binding sites in SOX21-bound chromatin, but not in SOX2-targeted regions (Figs. 4D, E and 6F, G). The AP-1 motif enrichment in SOX21-targeted chromatin was not attributable to cross-reactivity of the antibodies used in the ChIP-seq experiments (Supplementary Fig. 6H, I). Moreover, apart from those of SOX and AP-1, no other strongly enriched transcription factor binding motifs could be detected across the SOX21 peak regions (Fig. 4F).

Analysis of SOX21 peak regions revealed a strong co-enrichment of SOX and AP-1 motifs within 1–2 base pairs (Fig. 4G, H). By comparison, SOX2-targeted chromatin regions were preferentially enriched for SOX and POU motifs, spaced 2-3 base pairs apart (Supplementary Fig. 6J, K). Consistent with this observation, co-immunoprecipitation experiments confirmed a physical interaction between SOX21 and the AP-1 family member c-JUN, a highly expressed AP-1 family member in GPCs (Fig. 4I, J and Supplementary Tables 1, 2). To further examine the functional interplay between SOX21 and AP-1, we performed c-JUN ChIP-seq experiments in JM11 and JM13 GPCs. These analyses revealed that approximately 50% of the SOX21-bound chromatin regions overlapped with c-JUN occupancy (Fig. 4K–M). In contrast, SOX2 and c-JUN exhibited no notable binding overlap (Fig. 4K–M). Together, these binding analyses indicate that SOX21 promotes an anti-tumorigenic gene expression profile by targeting AP-1-bound chromatin regions.

### SOX21 represses AP-1-activated gene expression in GPCs

Given the central role of AP-1 transcription factors in the maintenance and progression of various types of cancer stem cells, we next investigated the gene regulatory interplay between SOX21 and AP-1. A comparison of genes bound by SOX21 (closest transcriptional start sites) and those regulated by SOX21 revealed that SOX21 predominantly functions to repress targeted genes in JM11 and JM13 GPCs (Fig. 5A), which contrasts with SOX2 that is mainly associated with genes activated by its overexpression (Fig. 5A). Interestingly, by comparing gene expression changes caused by pharmacological AP-1 inhibition (via the small molecules T-5224 [29] and SR 11302 [30, 31]) with those repressed by SOX21, we observed a strong overlap among genes suppressed by AP-1 inhibition and those downregulated by SOX21, which was not found among the genes activated by AP-1 inhibition (Fig. 5B, C). Consistent with this gene regulatory overlap, gene-set enrichment analysis (GSEA) showed a reduction of “*E2F targets*” and a positive enrichment of “*P53 pathway*” in JM11 and JM13 both in the presence of AP-1 inhibitors (T-5224) and upon DOX-induced SOX21 expression (Fig. 5D, E).

Based on the observation that SOX21 represses AP-1-driven gene expression, we aimed to identify the underlying molecular mechanisms. To address this issue, we employed ATAC-seq [32] to examine how SOX21 induction for 48 h alters chromatin accessibility at SOX21- and c-JUN-targeted regions. We also performed ChIP-seq experiments to assess their association with the epigenetic markers H3K4me1 and H3K27ac, indicative of primed and active enhancers, respectively [33, 34]. Quality control measures confirmed the reliability and reproducibility of the ATAC-seq experiments (Supplementary Fig. 7A–C). Principal component analysis of ATAC-seq data showed that DOX-induced SOX21 expression significantly altered the chromatin architecture, separating SOX21-induced samples from controls (Supplementary Fig. 7D). We identified approximately 50,000 chromatin regions with altered accessibility following SOX21 induction, with nearly equal numbers exhibiting increased or decreased ATAC-seq signals (Supplementary Fig. 7E and Supplementary Table 5). Notably, the accessibility of the chromatin regions commonly identified in the SOX21 and c-JUN ChIP-seq data decreased significantly (*p* = 1.3 × 10^–33^) upon SOX21 induction (Fig. 5F, I, J). Moreover, the reduction in accessibility, in response to SOX21 expression, was followed by a strong decrease in H3K27ac and H3K4me1 levels (*p* = 4.2 × 10^–215^ and *p* = 8.0 × 10^–107^, respectively) within SOX21 and c-JUN targeted chromatin (Fig. 5G–J). These findings demonstrate that the capacity of SOX21 to inhibit AP-1-driven gene expression in GPCs is associated with SOX21-mediated epigenetic inactivation of commonly targeted enhancer regions.

Our ChIP-seq analysis indicates that c-JUN is present at commonly targeted chromatin regions before SOX21 induction (Fig. 5I, J). To further correlate SOX21 binding with that of AP-1, we performed transcription factor footprint analysis on ATAC-seq data, a computational method to predict direct transcription factor binding at specific motifs. Comparing chromatin regions identified in the SOX21 ChIP-seq data with control regions containing a similar number of SOX and AP-1 motifs, we mostly observed strong enrichment of AP-1 footprints at SOX21 chromatin targets, an enrichment that was independent of SOX21 induction (Fig. 5K, L). Consistently, SOX footprints were mainly found upon DOX-induced SOX21 expression at chromatin regions preoccupied by AP-1 transcription factors (Fig. 5K). These findings suggest that SOX21 binding and subsequent repression of AP-1-driven gene expression are guided by pre-existing AP-1 occupancy.

### Overlapping functions of SOX21 and pharmacological AP-1 inhibitors in GPCs

Given that SOX21 represses AP-1-driven gene transcription, we next compared their activities in GPCs. To proceed, we examined the effects of AP-1 inhibition on JM11 and JM13 GPC survival, proliferation, and sphere-forming capacity. Inhibition of AP-1 function, with either T-5224 or SR 11302, significantly reduced GPC viability (Figs. 6A, B and 8A), as evidenced by a decreased fraction of EdU-incorporating cells, increased LDH-release and induction of apoptotic marker expression (Figs. 6C–E and 8B–D). In addition to impairing cell survival and proliferation, AP-1 inhibition markedly reduced sphere-forming capacity of JM11 GPCs, indicating loss of its self-renewal potential (Fig. 6F–H). Thus, inhibition of AP-1 function largely recapitulates the activity of SOX21 in GPCs.

To test the reciprocal relationship, we performed RNA-seq analyses after enforced c-JUN expression through lentiviral transduction. These experiments revealed that genes upregulated by c-JUN in JM11 and JM13 GPCs, but not those repressed, were highly enriched among genes downregulated by SOX21 (Figs. 6I and 8E). Consistent with this result, forced c-JUN expression effectively rescued the SOX21-induced reduction in GPC viability and proliferation (Figs. 6J–L and 8F, G). Together, these data demonstrate that the tumor-suppressive effects of SOX21 require repression of AP-1 signaling and position SOX21 as a negative regulator of AP-1-driven oncogenic programs in GPCs.

## Discussion

The prevailing treatment protocol for GBM consists of surgical resection followed by radiation and chemotherapy with DNA-alkylating agents, such as TMZ [18, 21]. However, this approach often fails due to its inability to eradicate treatment-resistant GPCs. For instance, this treatment regime does not elevate the gene expression of SOX21 [35], which we demonstrated efficiently sensitizes GPCs to TMZ. Therefore, the development of novel therapeutic strategies that simultaneously target both the bulk tumor cells and GPCs is of critical importance.

By generating patient-derived GPCs, equipped with an inducible SOX21 expression system, we have shown an inverse correlation between SOX21 expression levels and GPC self-renewal and survival. The reduction in GPC properties following DOX-induced SOX21 expression was accompanied by a significant decrease in the expression of GPC-related genes such as *SOX2*, *HDAC9*, *CDK6*, and *EFNB2*, all of which have been implicated in GBM pathogenesis [23–27]. Previous loss-of-function studies in mice models have demonstrated that SOX21 is essential for preventing malignant transformation of brain neural stem cells exposed to an oncogenic insult [16]. Moreover, experiments in which SOX21 is overexpressed in primary human GPCs before transplantation into mice highlight the potential of SOX21 to inhibit their tumor-initiating capacity [16, 17]. While these results align with the tumor suppressor function of SOX21, their therapeutic relevance can be questioned. In the present study, we demonstrated the ability of SOX21 to impede tumor progression upon its induced expression in established GBM, which ultimately leads to an extended survival of the orthotopically transplanted mice. These results suggest that DOX-induced SOX21 expression suppresses tumor growth by reducing the pool of proliferative GPCs. It is worth noting that high concentrations of DOX alone have previously been reported to negatively affect GPC proliferation [36]. However, the tumor-suppressive effect observed in animals with DOX-induced SOX21 expression was significantly stronger than in DOX-treated control mice, highlighting a SOX21-specific effect beyond any impact of doxycycline alone. Thus, collectively our functional analyses demonstrate that elevating SOX21 levels in established GBM reduces tumor growth and prolongs survival, which agrees with clinical observations showing that higher SOX21 expression correlates with improved GBM patient survival. Interestingly, the tumor suppressor activity of SOX21 appears to be context-dependent. Opposite to the activity in GPCs, SOX21 has been reported to promote cell proliferation and survival of both colon and pancreatic cancer cells [37, 38]. Consistently, the survival of colon cancer and pancreatic cancer patients is negatively correlated with SOX21 expression levels [37, 38]. In colon cancer cells SOX21 appears to achieve its tumor promoting activity by inducing the transcription factor POU4F2, an effect not observed in GPCs (Supplementary Table S3).

In this study, we identified a strong inverse functional correlation between SOX21 and AP-1 proteins. AP-1 transcription factors have, opposite to the tumor suppressor functions of SOX21, been identified as critical drivers of cancer stem cell proliferation, survival, aggressiveness, and treatment resistance [9, 39, 40]. While high SOX21 expression levels correlate with improved GBM patient outcomes, elevated levels of AP-1 transcription factors have been reported to predict poorer prognosis [9]. We propose that SOX21 suppresses GPCs by counteracting AP-1-regulated pathways. This conclusion is supported by the identification of a physical interaction between SOX21 and c-JUN in GPCs, as well as the strong overlap between c-JUN and SOX21 targeted chromatin regions. Moreover, pharmacological inhibition of AP-1 in GPCs significantly phenocopied the effects of SOX21 induction, including the downregulation of key genetic pathways and a reduction in GPC proliferation, survival, and tumor-sphere formation. Conversely, forced expression of c-JUN in the presence of SOX21 counteracted SOX21-mediated gene repression and rescued GPC viability and proliferation. However, despite clearly demonstrating that SOX21 negatively regulates AP-1 activity, it cannot be ruled out that SOX21 and AP-1 partly control GPC self-renewal and viability through unique molecular mechanisms.

How is SOX21 counteracting the AP-1 function in GPCs? We show that SOX21 binding is preferentially directed to chromatin regions that are accessible and pre-bound by AP-1. AP-1 transcription factors promote chromatin opening through an interaction with the SWI/SNF chromatin remodeling complex [40–42], a function that can contribute to tumor initiation [40]. In contrast, the binding of SOX21 leads to reduced accessibility of targeted chromatin and a strong decrease in the presence of H3K27ac and, to a lesser extent, of H3K4me1, indicating a transition from an active to an inactive or poised enhancer state [34]. Hence, SOX21 may suppress GPC maintenance and proliferation by limiting the capacity of AP-1 to facilitate enhancer accessibility and possibly the binding of additional factors, driving tumor-promoting gene expression.

In contrast to SOX21, SOX2 has been referred to as an oncogenic factor due to its essential role in the maintenance and expansion of cancer stem cells [12, 27, 43–47]. In gliomas, elevated SOX2 expression correlates with increased tumor aggressiveness and poor patient prognosis [48]. Although induced SOX2 expression did not alter GBM growth pattern in our model, previous knockdown studies have confirmed its necessity for GPC proliferation and tumor-inducing capacity [26, 27]. Structurally, the DNA-binding high-mobility group (HMG) domains of SOX21 and SOX2 are highly conserved; however, while SOX21 functions as a transcriptional repressor, SOX2 acts as a transcriptional activator [49]. Interestingly, a modified version of SOX2, engineered to function as an epigenetic repressor, exhibits similar tumor-suppressive properties as SOX21, effectively counteracting GBM progression when delivered via viral vectors into the brains of mouse tumor models [50]. Although one interpretation of these findings aligns with the previous idea that SOX21 and SOX2 act on a shared set of target genes [15, 51], we found a surprisingly limited target site overlap (<10%) in GPCs. Consequently, induced SOX21 expression regulated an insignificant fraction of the SOX2-bound genes in GPCs. One explanation for the divergent binding pattern of SOX21 and SOX2 is that the target selection of SOX proteins is generally dependent on their collaboration with partner transcription factors [51]. Here, we demonstrated that while SOX21 physically interacts with c-JUN and binds in the vicinity of AP-1 transcription factors, SOX2 binding is enriched around motifs for POU transcription factors, which are well-established partner factors of SOX2 in different stem cell compartments [51–54]. These findings suggest that although SOX21 and SOX2 exert opposing effects on similar processes in GPCs, they achieve these functions by binding distinct sets of target genes in cooperation with unique partner factors.

This study identifies SOX21 as a key tumor suppressor that inhibits AP-1-driven oncogenic signaling in GPCs. While direct pharmacological activation of SOX21 is currently not feasible, our data show that targeting pathways functionally linked to SOX21 represents a promising therapeutic strategy. In addition, gene-delivery approaches, such as viral vectors enabling SOX21 expression within tumor cells and the surrounding brain parenchyma, could offer a complementary strategy. According to the findings presented in this study, such approaches may have the advantage of simultaneously reducing AP-1 activity and increasing GPC sensitivity to TMZ.

By defining the molecular mechanisms through which SOX21 controls glioma biology, this work provides new insight into the regulatory networks that maintain glioblastoma stemness. These findings may inform the development of future therapeutic interventions aimed at preventing recurrence in glioblastoma.

## Material and methods

See Supplementary Material and Methods for additional details.

### Antibodies

Primary antibodies: SOX21 (R&D, AF3538); SOX2 (Seven Hills Bioreagent, WRAB-1236); cJUN (Invitrogen, #702170); OLIG2 (Millipore AB9610); (HuNu (Abcam, ab190710); KI67 (ThermoFisher, 14-5698-82; Abcam, ab16667); FLAG (Abcam, ab205606); HA (Santa Cruz, sc-7392); H3K27Ac (Diagenode, C15410174); H3K4me1 (Diagenode, C15410194). BIM (Cell Signaling Technology, #2933), BAK (Cell Signaling Technology, #12105), BAX (Cell Signaling Technology, #2772), CASPASE3 (Cell Signaling Technology, #9665), cleaved CASPASE3 (Cell Signaling Technology, #9664), GPX4 (Abcam, ab 125066, 1:1000), LC3B (Sigma, L7543, 1:1000), ß-actin (Abcam, ab20272), anti-rabbit IgG H&L-HRP (Abcam, ab97015).

Secondary antibodies: Donkey anti-Goat-488 (Alexa Fluor Invitrogen, A11055); Donkey anti-Rat-488 (Alexa Fluor Invitrogen, A2120); Donkey anti-Rabbit-555 (Alexa Fluor Invitrogen, A31572); Donkey anti-Mouse-555 (Alexa Fluor Invitrogen, A31570); Donkey anti-Goat-555 (Alexa Fluor Invitrogen, A21432); Donkey anti-Rabbit-488 (Alexa Fluor Invitrogen, A21206).

### GPCs cultures

Human glioblastoma (GBM) samples were obtained from Karolinska Institute Biobank (Ethical permit 2023-01366-01). GPCs were isolated and cultured on poly-L-Ornithine (Sigma Aldrich)/Laminin (Sigma Aldrich)-coated plates in Human NeuroCult NS-A Proliferation media (STEMCELL Technologies) supplemented with 2 μg/mL heparin (STEMCELL Technologies), 10 ng/mL FGF (STEMCELL Technologies), 20 ng/mL EGF (STEMCELL Technologies), and penicillin/streptomycin (Gibco). The AP-1 inhibitors SR11302 (ChemCruz) and T-5224 (Cayman Chemical) were used at a concentration of 10 μM and 75 μM, respectively [40].

### Generation of viral expression vectors and virus production

The coding sequences of SOX2 and SOX21 were amplified from human genomic DNA (PCR-261, Jena Bioscience), including a 3xFLAG tag in the 3´amplifying PCR primers. SOX2-FLAG and SOX21-FLAG PCR products were subcloned into the BamH1 site of the pLVX-TetOne-Puro Vector (Takara Bio). The human c-JUN coding sequence was amplified by PCR from the vector (p6600 MSCV-IP N-HAonly JUN; Addgene plasmid #34898). The PCR fragment was inserted into pLVX-EF1α-IRES-mCherry. All constructs were confirmed by Sanger sequencing. Lentiviral vector pLenti-PGK-V5-LUC (w528-1) (plasmid #19166) was obtained from Addgene. Viruses were packaged in 293FT cells with either second- or third-generation packaging systems.

### SOX21 and SOX2 expressing GPCs

To engineer GPCs with SOX21 and SOX2 expression systems, primary GPCs (JM11, JM12 and JM13) were transduced with DOX-regulated lentiviruses (pLVX-SOX21, pLVX-SOX2, or pLVX). Transduced cells were selected with 6 μg/mL Puromycin (Gibco) for 4 weeks. For in vivo experiments, cells were further transduced with a constitutive Luciferase-expressing lentivirus (pGK-Luc). All primary cells were kept below 10 passages and tested for mycoplasma using MycoAlert Mycoplasma Detection kit (Lonza). DOX (200 ng/mL) was added for 48 h pre-assay unless otherwise specified.

### Establishment and analysis of orthotopic GBM models

Animal experiments adhered to Swedish animal welfare laws (Dnr 3696-2020). Human transgenic GPCs (JM11; *pLVX, pLVX-SOX21, pLVX-SOX2*, JM12; *pLVX, pLVX-SOX21, pLVX-SOX2*; JM13; *pLVX, pLVX-SOX21*) (150,000 cells per injection) were prepared at 50,000 cells/μL with >80% viability and maintained on ice for up to two hours before transplantation. Stereotactic injections targeted the striatum in immunocompromised NOD.CB17-PrkcSCID/J, 6–10 weeks old, female mice at 2.0 mm lateral, 1.0 mm anterior, and 2.5 mm depth from bregma (3 µl). Pre-operative analgesia (0.1 mg Norocarp/Carprofen (Zoetis), 2–5 mg/kg) was administered subcutaneously, and anesthesia was maintained with 2-3% isoflurane (Zoetis) in O^2^ (1 L/min) during transplantation. Local analgesia (2.5 mg/mL, Marcain, Aspen Pharmacare) was applied at the incision site. Postoperative care included subcutaneous administration of Temgesic/Vetergesic (0.05–1 mg/kg) (Ceva Santé Animale) for two days, with daily monitoring for one week. Mice exhibiting distress were euthanized according to ethical guidelines. For the induction of transgenic SOX21 and SOX2 expression, mice were provided with DOX-containing food (0625 g/kg; SAFE-lab).

### In vivo bioluminescence imaging

Tumor establishment and progression were assessed using IVIS Spectrum CT Imaging (Perkin Elmer). Mice received intraperitoneal injections of 150 mg/kg D-Luciferin (Promega) and were imaged 23 min post-injection to capture peak luminescence. Animals were maintained at 37 °C under gaseous anesthesia (2–3% isoflurane in 1 L/min O^2^) during imaging. Data were processed in Living Image software (PerkinElmer). Tumor progressions were scored every second week. Survival data were recorded for statistical analysis.

### Tissue processing and histology

Mice were deeply anesthetized with avertin (Sigma-Aldrich) before transcardial perfusion with PBS (Gibco) followed by 4% paraformaldehyde (Sigma-Aldrich). Brains were extracted, post-fixed in paraformaldehyde, and processed for cryopreservation or paraffin embedding. Coronal sections (5–15 μm) were prepared. Tumor morphology was assessed via hematoxylin and eosin (H&E) staining (Histolab). Images were acquired using a Zeiss AxioScan.Z1 slide scanner and analyzed with QuPath (v0.2.3) and ImageJ.

### Immunofluorescence

Tissue sections underwent deparaffination, antigen retrieval (Dako) and blocking with 4% donkey serum (Jackson ImmunoResearch) or FBS. Primary antibodies were incubated overnight at room temperature, followed by secondary antibody labeling. Nuclei were counterstained with DAPI. Images were acquired using a ZEISS LSM700 confocal microscope and analyzed with ImageJ.

### Neurosphere formation assay

Primary GPCs were dissociated into single-cell suspensions and seeded into 24-well low-attachment plates (1000 cells/well) with DMEM/F12-Glutamax (Gibco), N-2 (Gibco), B-27 (Gibco), recombinant human EGF, bFGF, 1.5% FBS (Gibco), and 0.05% BSA (Sigma Aldrich). Rock-inhibitor/Y-27632 (1 µM, STEMCELL Technologies) was added for the first 1–2 days. DOX (250 ng/mL) or the AP-1 inhibitors SR11302 (10 μM; ChemCruz) and T-5224 (100 μM, Cayman Chemical) were applied on day three. N-2, B-27, EGF, bFGF, DOX or AP-1 inhibitors were re-supplied every two days. Spheroid growth was documented after 10 days (DOX) or 14 days (AP-1 inhibitors) using a Thermofisher EVOS M7000 microscope and analyzed using ImageJ.

### Cell imaging, proliferation and assays

Cells were imaged using the EVOS cell imaging system (10x magnification). Proliferation was measured with a Click-iT EdU Flow Cytometry Assay Kit (Invitrogen) and analyzed by flow cytometry (BD FACSCanto II, 50,000 events/sample). Apoptosis was assessed using FITC Annexin V detection (BD Biosciences) and analyzed by FACS (BD FACSCanto II). Data were processed using FlowJo software.

### Western blot and Co-IP

Protein lysates were prepared using RIPA buffer (Sigma Aldrich) supplemented with a protease inhibitor mixture (Roche). Cells were lysed on ice for 30 min with intermittent vortexing, followed by centrifugation at 14,000 × *g* for 10 min at 4 °C to clear debris. Protein concentrations were determined using a Bradford Assay (Bio-Rad).

For Western blot, equal amounts of protein lysates were mixed with loading buffer, denatured at 95 °C for 5 min, and resolved on an 8–12% SDS-PAGE gradient gel alongside a protein ladder (ThermoScientific). Proteins were transferred onto a nitrocellulose membrane (Amersham) and blocked in 5% milk in PBS/0.1% Tween-20 (Sigma) for 1 h. Membranes were incubated overnight at 4 °C with primary antibodies at optimized dilutions. Secondary HRP-conjugated antibodies (Vector Laboratories) were applied at a 1:5,000 dilution, followed by detection with ECL substrate (ThermoScientific).

For Co-IP, cells were lysed in a buffer containing 50 mM Tris-HCl (pH 7.4), 150 mM NaCl, 1% NP-40, and protease/phosphatase inhibitors. Lysates were cleared by centrifugation at 12,000 × *g* for 15 min at 4 °C. 50 U of DNase I (ThermoScientific) per 1 mL of lysate was added and incubated at 37 °C for 20 min. Immunoprecipitation was performed with 5 µg anti-HA or control IgG conjugated to Dynabeads (ThermoScientific) overnight at 4 °C. Beads were washed three times, and bound proteins were eluted with SDS sample buffer. Proteins were separated by SDS-PAGE and detected by immunoblotting using anti-FLAG and anti-HA antibodies to assess interactions between SOX21 and c-JUN. Uncropped western blots are presented in the Supplementary Materials.

### Statistical analysis

Significance of cell/sphere numbers or sphere sizes was determined using unpaired t-tests (two-tailed). Cell marker expression in human GBM specimens was analyzed using Fisher’s exact test. Kaplan-Meier survival curves were analyzed using log-rank (Mantel-Cox) tests with or without adjusted Cox regression. For gene set enrichment, simple chi-square tests were used. ATAC-seq, H3K27ac and H3K4me3 ChIP-seq signals in GPCs with or without DOX-induced SOX21 expression were analyzed with Welsh test and unpaired t-test. Data are presented as mean or p-value and barographs as ± SEM. A *p*-value < 0.05 was considered significant. Sample sizes were ≥ 3 to meet statistical criteria for comparisons. The variance between samples derived from comparable biological experiments was not substantial, and therefore, formal tests for equality of variances were not performed.

## Supplementary information

Supplementary Figures

Supplementary Methods

Supplementary Table 1

Supplementary Table 2

Supplementary Table 3

Supplementary Table 4

Supplementary Table 5

Supplementary WB data

## Data Availability

The data generated in this study, Raw fastq files from ChIP-seq, RNA-seq, and ATAC-seq experiments, are available via NCBI BioProject ID PRJNA1128363.
