# Supplementary material for: SOX21 suppresses glioblastoma growth by repressing AP-1 activity
Source: Cell Death Dis. 2026 Jan 31;17(1):191. doi: 10.1038/s41419-026-08442-5 (PMC12876893; doi:10.1038/s41419-026-08442-5)
Supplement: Supplementary file 1 — Supplementary Figures [file 41419_2026_8442_MOESM1_ESM.pdf]

Supplementary Fig. 1

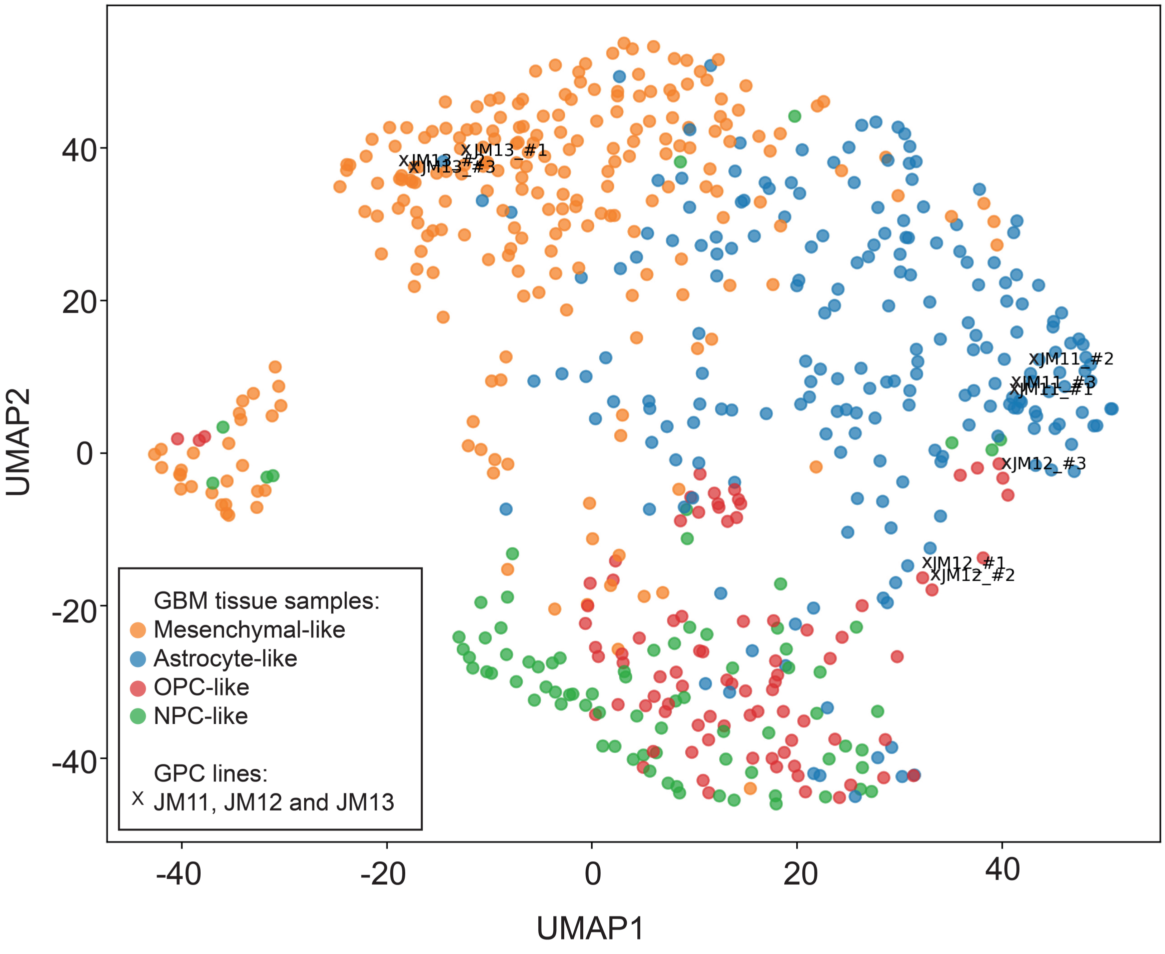

Supplementary fig. 1: Molecular Subtype Specification of Primary GPCs.

UMAP (Uniform Manifold Approximation and Projection) was used to analyse RNA-seq data from GPC lines (JM11, JM12, and JM13; replicates #1–3) and 539 TCGA GBM tissue samples. This analysis classifies JM11 GPCs as Astrocyte-like (blue), JM12 GPCs as oligodendrocyte-progenitor-like (OPC-like) (red), and JM13 GPCs as mesenchymal-like (yellow).

Supplementary Fig. 2

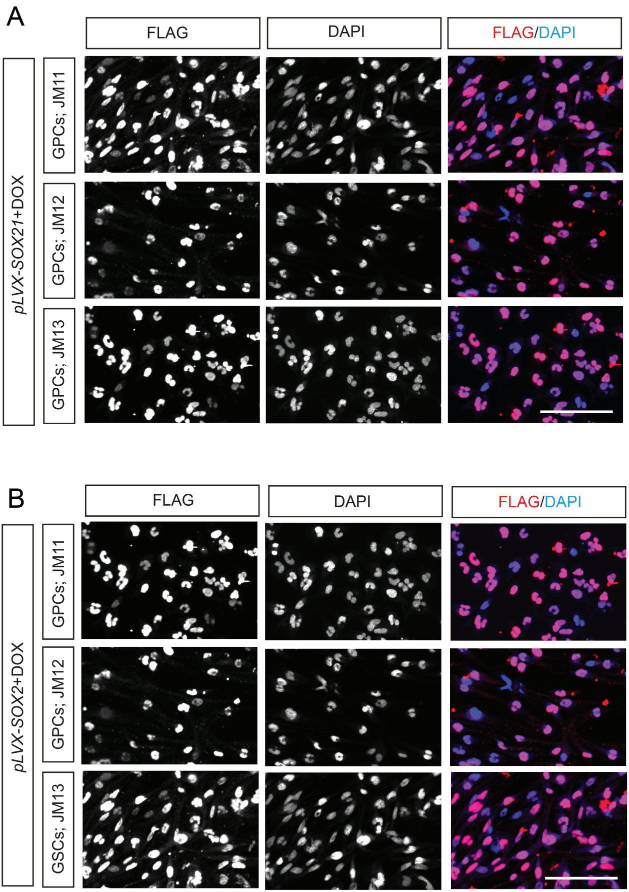

Supplementary fig. 2: Immunohistochemical Validation of Inducible SOX21 and SOX2 Expression Systems.

A and **B** Immunohistochemical analysis demonstrating the efficiency of FLAG-tagged SOX21 (**A**) and SOX2 (**B**) induction in GPCs (JM11, JM12 and JM13) following 48 hours of DOX treatment. Scale bar: 30 μm.

Supplementary Fig. 3

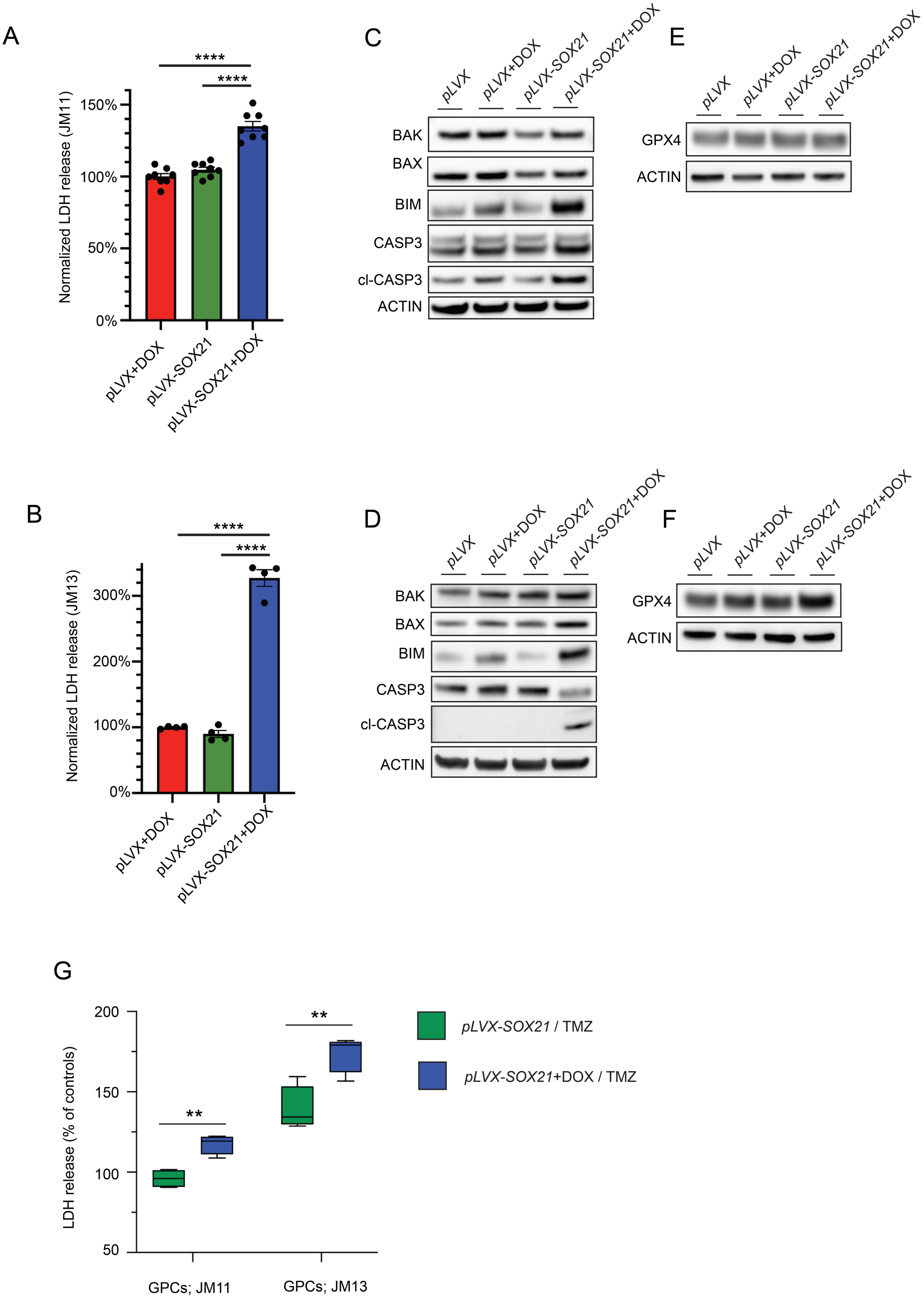

Supplementary fig. 3: SOX21 induce apoptosis and sensitizes GPCs to TMZ.

A, **B** Bar graphs show that LDH release is significantly increased in JM11 and JM13 GPCs after DOX-induced SOX21 expression (blue) for 72 to 96 hours, in comparison with GPCs cultured under control conditions (red and green). **C**, **D** Western blot analyses show an upregulation of the pro-apoptotic markers BAK, BAX, BIM and of the definitive apoptotic markers total and cleaved CASPASE3 in JM11 (**C**) and JM13 (**D**) GPCs following induced SOX21 expression. **E**, **F** Western blot analyses show no or only weak upregulation of the ferroptosis marker GPX4 in JM11 (**E**) and JM13 (**F**) GPCs following induced SOX21 expression. **G** Normalized LDH release values in GPCs treated with TMZ (175 μM) alone (green) or in combination with induced SOX21 expression (blue).

Supplementary Fig. 4

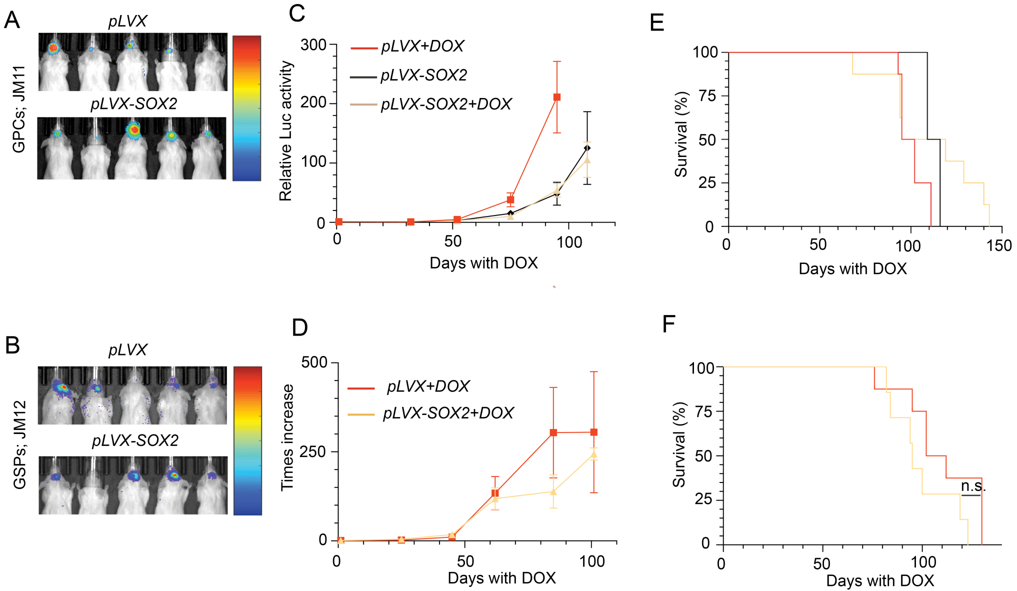

Supplementary fig. 4: Impact of Induced SOX2 Expression on GBM Progression in Mice.

A, B Bioluminescence imaging confirming tumor establishment prior to DOX administration in mice transplanted with control or SOX2-inducible JM11 (**A**) or JM12 (**B**) GPCs. **C, D** Tumor growth curves showing luciferase-based quantification of tumor burden in mice transplanted with control (red lines) or SOX2-inducible (yellow and black lines) JM11 (**C**) or JM12 (**D**) GPCs after receiving control or DOX-supplemented food. **E, F** Kaplan-Meier analyses show the survival of mice transplanted with control (red) or SOX2-inducible (black and yellow) JM11 (**E**) or JM12 (**F**) GPCs after receiving control or DOX-supplemented food.

Supplementary Fig. 5

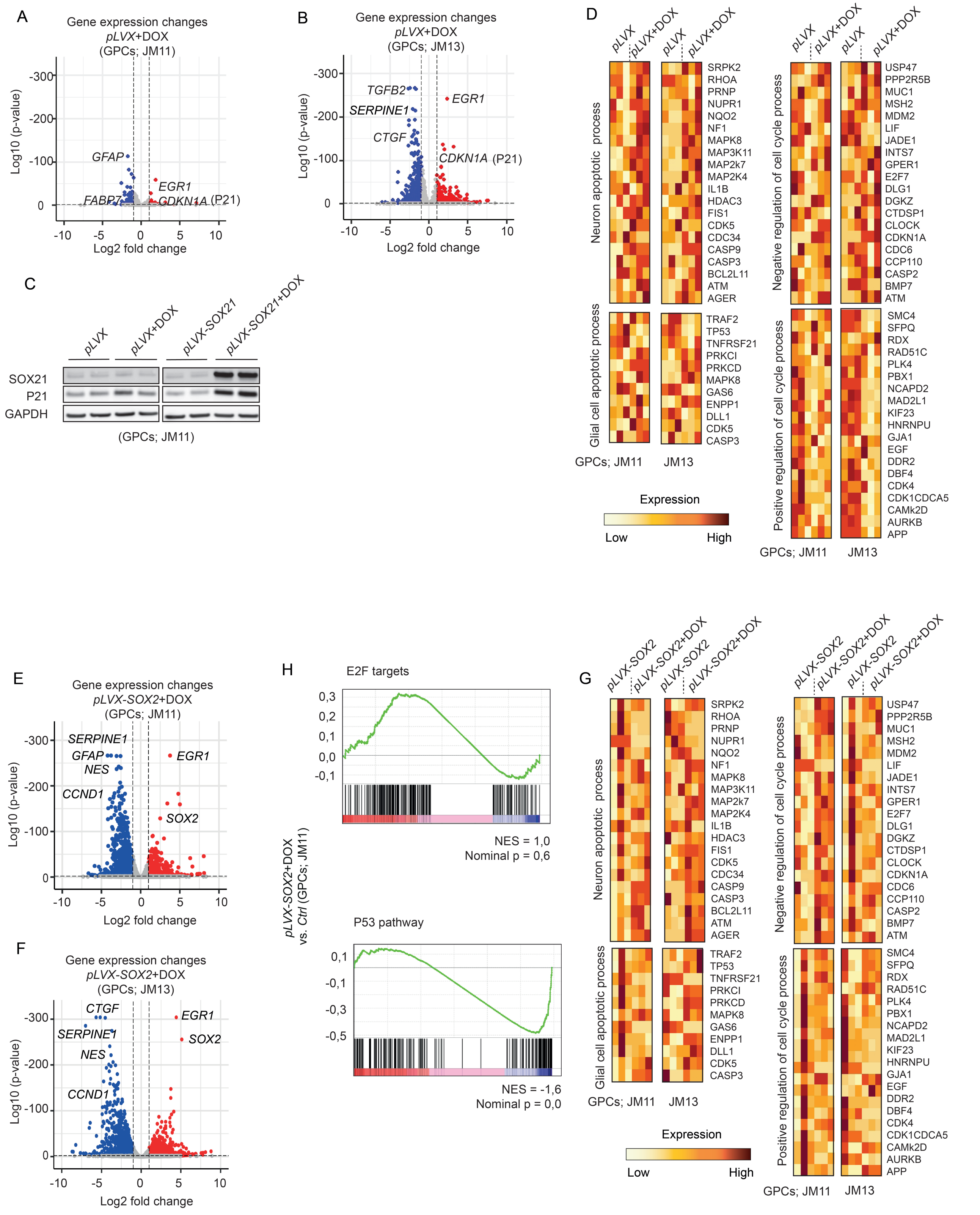

Supplementary fig. 5: Gene Regulation induced by DOX and SOX2 in GPCs.

A, **B** Volcano plots display differential gene expression in control JM11 (**A**) and JM13 (**B**) GPCs after 48 hours of DOX treatment. Upregulated genes (red) and downregulated genes (blue) are shown (false discovery rate < 0.01). **C** Western blot analysis aligns with transcriptome data and shows a substantial upregulation of P21 protein in JM11 GPCs following DOX-induced SOX21 expression. **D** Heatmaps show the regulation of gene sets and their linked GO-terms in control GPCs (*pLVX*), cultured with or without DOX for 48 hours. **E**, **F** Volcano plots show differentially expressed genes in SOX2-inducible JM11 (**E**) and JM13 (**F**) GPCs (*pLVX-SOX2*), cultured with DOX for 48 hours. Genes up- and downregulated, in comparison to controls cells cultured without DOX, are represented with red and blue dots, respectively (false discovery rate < 0,01). **G** GSEA of differentially regulated genes in GPCs following SOX2 induction for 48 hours (*pLVX-SOX2*+DOX vs. *pLVX-SOX2*). **H** Heatmaps show the expression of gene sets, and their linked GO-terms in SOX2-inducible GPCs cultured with or without DOX for 48 hours.

Supplementary Fig. 6

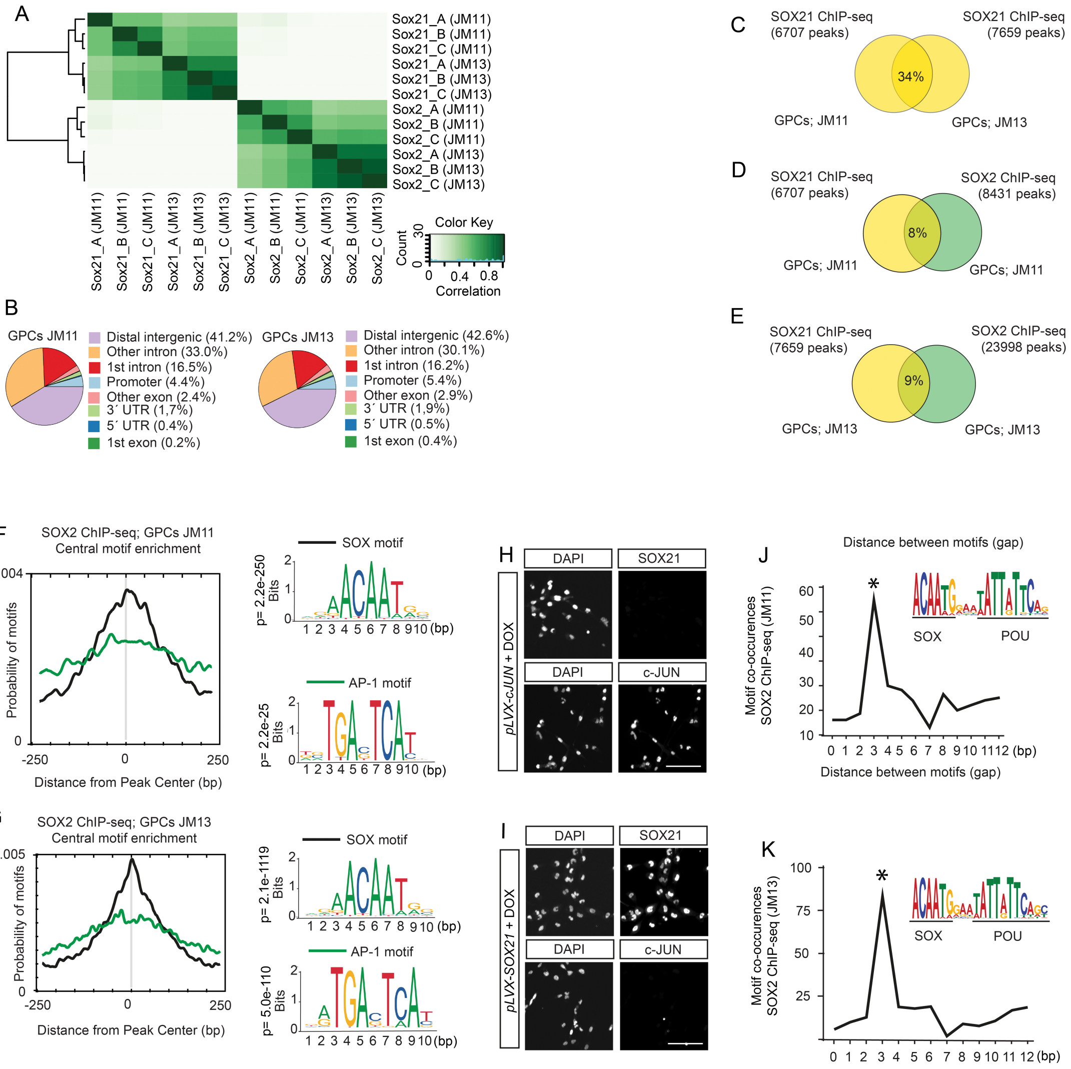

Supplementary fig. 6: Characterization of SOX21 and SOX2 Targeted Chromatin Regions in GPCs.

A Heatmap with an associated dendrogram shows the clustering and cell-type specific similarities between SOX21 and SOX2 ChIP-seq replicates in JM11 and JM13 GPCs. **B** Diagrams show the distribution of genomic features across SOX21 ChIP-seq peak regions identified in JM11 and JM13 GPCs. **C** Venn diagrams comparing SOX21 ChIP-seq peak regions in JM11 and JM13 GPCs, based on three independent replicates. **D, E** Venn diagrams comparing SOX21 and SOX2 ChIP-seq peak regions in JM11 (**D**) and JM13 (**E**) GPCs. **F, G** Enrichment of centrally positioned SOX motifs (black) and AP-1 motifs (green) in SOX2 ChIP-seq peaks in JM11 (**F**) and JM13 (**G**) GPCs, with motif distances to the center of SOX2 peaks measured in base pairs (bp). P-values of best-matching SOX and AP-1 motifs are shown. **H**, **I** GPCs overexpressing c-JUN (**H**), and SOX21 (**I**) stained with SOX21 and c-JUN antibodies. Results confirm the specificity of the SOX21 antibodies used in the SOX21 ChIP-seq experiments. Scale bar: 30 µm. **J**, **K** Graphs depicting the most significant motif spacing between SOX and POU motifs in SOX2 ChIP-seq peaks detected in JM11 (**J**) and JM13 (**K**) GPCs. Distance calculations are based on nucleotide spacing between the last base of the SOX motif and the first base of the POU motif.

Supplementary Fig. 7

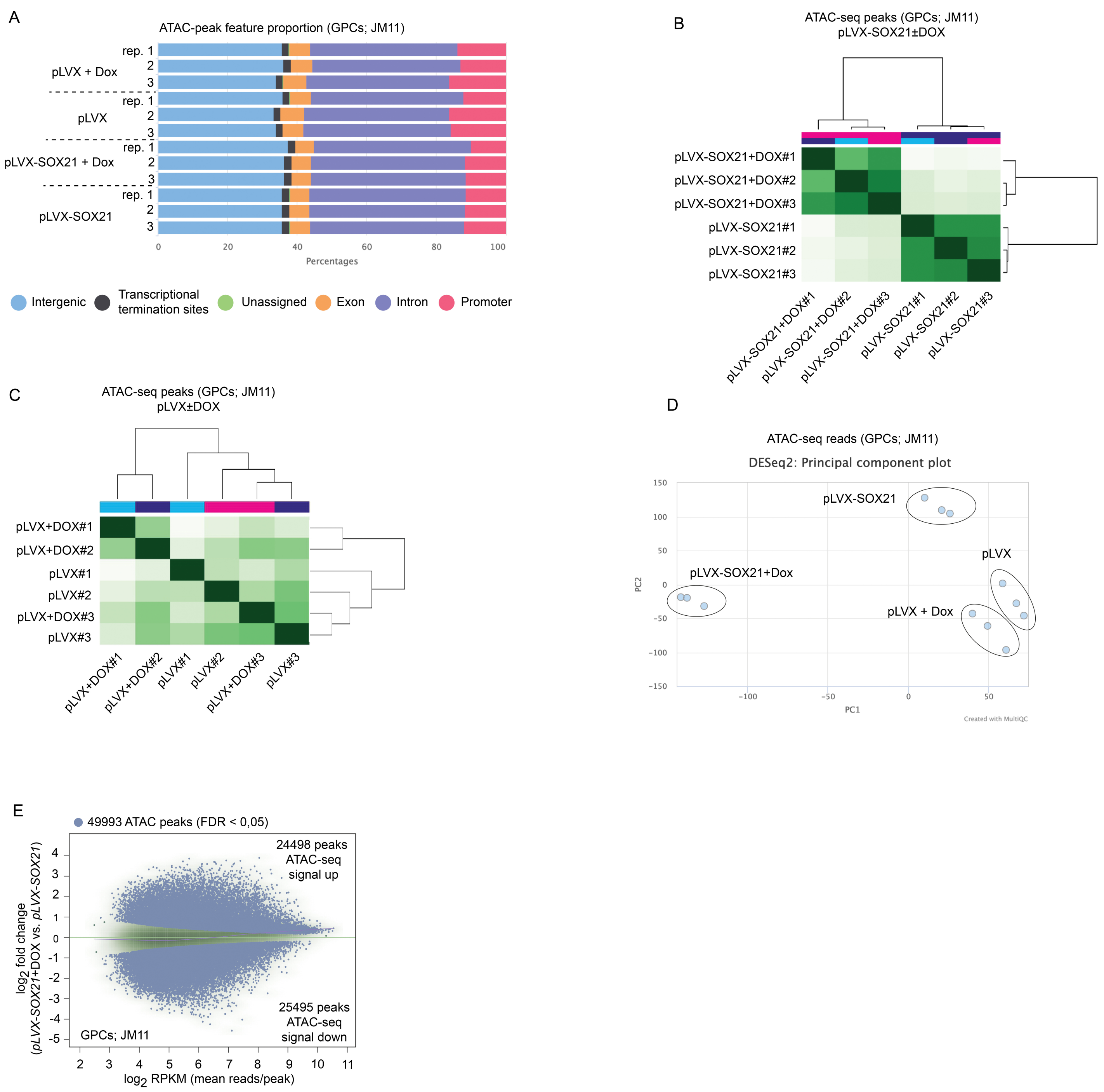

Supplementary fig. 7: Quality Control Measures for ATAC-seq Experiments.

A Bar graph showing the distribution of genomic features associated with ATAC-seq peaks in *pLVX* and *pLVX-SOX21* JM11 GPCs, cultured with or without DOX for 48 hours. Biological replicates for each condition are shown. **B**, **C** Heatmaps with corresponding dendrograms show similarities between ATAC-seq peak profiles in SOX21-inducible (**B**) and control (**C**) JM11 GPCs, cultured with or without DOX 48 hours. **D** Principal component analysis of ATAC-seq read counts in *pLVX* and *LVX-SOX21* JM11 GPCs, cultured with or without DOX 48 hours. Replicates for each condition are encircled, demonstrating clustering by genotype and treatment. **E** MA-plot showing changes in chromatin accessibility in JM11 GPCs, following 48 hours of DOX-induced SOX21 expression compared with untreated controls. Regions with significantly increased or decreased accessibility (FDR < 0.05) are highlighted in blue.

Supplementary Fig. 8

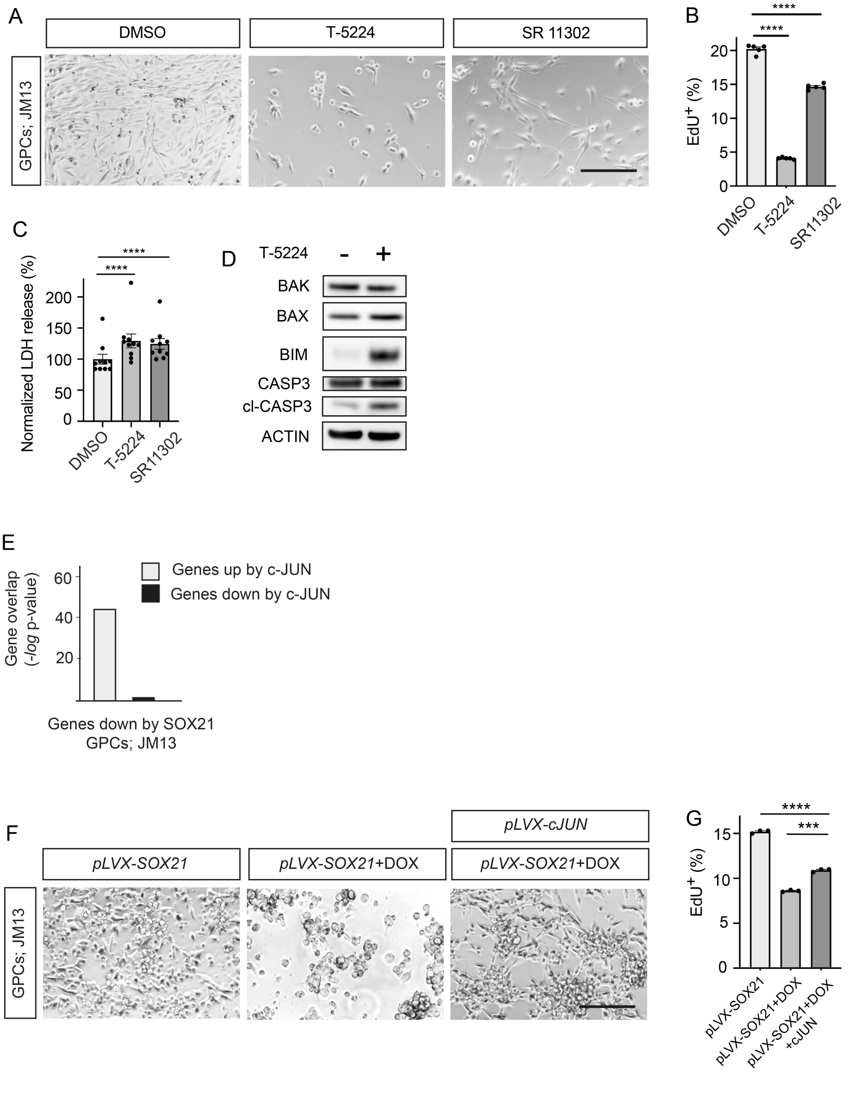

Supplementary fig. 8: c-JUN Counteracts SOX21 Mediated Growth Suppression in GPCs.

A Representative images of JM13 GPCs treated for 4 days with DMSO (control), T-5224, or SR 11302. Scale bar: 60 µm. **B**, **C** Quantification of cell proliferation (EdU incorporation) (**B**) and cytotoxicity (LDH release) (**C**) following AP-1 inhibition. **D** Western blot analysis showing an upregulation of apoptotic markers in JM13 GPCs treated with the AP-1 inhibitor (T-5224) for 48 hours. **E** RNA-seq analysis of JM13 GPCs showing strong enrichment of SOX21 downregulated genes among those upregulated by c-JUN (grey), but not among c-JUN downregulated genes (black). **F** Representative images showing that enforced c-JUN expression in JM13 GPCs for 4 days rescues the reduction in cell number caused by DOX-induced SOX21 expression. Scale bar, 60 µm. **G** Quantification of proliferation (EdU incorporation) following DOX-induced SOX21 expression either alone or in combination with forced c-JUN expression.
