# Supplementary material for: SOX21 suppresses glioblastoma growth by repressing AP-1 activity
Source: Cell Death Dis. 2026 Jan 31;17(1):191. doi: 10.1038/s41419-026-08442-5 (PMC12876893; doi:10.1038/s41419-026-08442-5)
Supplement: Supplementary file 2 — Supplementary Methods [file 41419_2026_8442_MOESM2_ESM.pdf]

Supplementary Materials

SOX21 Suppresses Glioblastoma Growth by Repressing AP-1 Activity

Eltjona Rrapaj *et al.*

* Corresponding author: Jonas Muhr, e-mail: jonas.muhr@ki.se

Supplementary Material and Methods

Gene Expression and Survival Analysis in TCGA and CGGA Glioblastoma Cohorts

RNA-seq raw counts (RSEM protocol) and clinical annotations for The Cancer Genome Atlas glioblastoma (TCGA GBM)[1] cohort were obtained from the Genomic Data Commons (GDC) data portal and the Xena platform[2]. Only patients with a primary glioblastoma diagnosis consistent with the 2021 WHO CNS Tumor Classification[3] were included (N=143). Gene counts were normalized using DESeq2, and patients were stratified into high and low SOX21 expression groups based on the cohort-specific median. The impact of SOX21 expression on overall survival was assessed using the log-rank test. For the CGGA cohort[4], RNA-seq count data from the Chinese Glioma Genome Atlas (CGGA) mRNAseq_325 and mRNAseq_693 batches were retrieved from the CGGA portal. Patients included in the analysis had primary, IDH-wildtype, WHO grade IV glioblastomas with non-codeleted 1p/19q status (N=155). To avoid bias due to differences in sequencing depth and platform between the two batches, each batch was normalized independently using DESeq2, and patients were stratified into high and

low SOX21 expression groups based on the batch-specific median. Overall survival analysis was performed using the log-rank test and batch adjusted Cox regression. All analyses were performed using R 4.4.0[5].

Temozolomide sensitivity assay

GPCs (pLVX-SOX21; JM11 and JM13) were seeded at 20,000 cells per well in 96-well plates. Attached cells were treated with temozolomide (SigmaAldrich) at final concentrations of 175 µM with or without the presence of DOX. DMSO were used as vehicle control in non-treated cells. After 72 hours of incubation, cytotoxicity was assessed using the CyQUANT™ LDH Cytotoxicity Assay Kit (Thermo Fisher Scientific) according to the manufacturer's instructions. All experimental conditions were performed in quadruplicate.

ChIP-Seq

ChIP experiments were conducted following established protocols using the following antibodies: goat anti-SOX21, rabbit anti-SOX2, rabbit anti-cJUN, rabbit anti-H3K4me1, and rabbit anti-H3K27Ac. ChIP libraries were prepared using the ThruPLEX DNA-Seq Kit (Takara) and sequenced on the Illumina NovaSeq6000 SP-100 platform, generating paired-end 2x50 bp or 2x150 bp reads, with a sequencing depth exceeding 2 ×10^7 reads per experiment.

ChIP reads were aligned to the human genome assembly hg19 using Bowtie (v1.3.1). Correlation plots between replicate BAM files were generated with deepTools (v2.5.1). MACS2 was used for peak calling under default settings, except for SOX2 and SOX21 ChIP in JM13, where an FDR threshold of 0.001 was applied. Consensus peak sets were defined by detecting signals across all three replicates. Blacklisted regions from hg19 (Encodeproject.org) were excluded. Centrally enriched motifs were identified with CentriMo, and spacing between DNA-binding motifs was analyzed with SpaMo (v5.5.5).

For cJUN, H3K27Ac and H3K4me1 ChIP-seq, reads were pooled from two replicates each. After duplicate removal, pooled files were used without further processing. To compare ChIP-seq data with ATAC-seq (mapped to hg38), peak regions were converted from hg19 to hg38 using LiftOver (UCSC). Peak overlaps (>50%) were considered significant using the BEDTools intersect function. SeqMINER was used to map cJUN-ChIP, H3K27Ac-ChIP, H3K4me1-ChIP, and ATAC-seq reads onto SOX21 peak regions.

Chromatin accessibility and histone modification profiles of SOX21 and c-JUN targeted region were analyzed using ATAC-seq data and ChIP-seq data for H3K27ac and H3K4me1. Signal matrices were processed in Python with pandas, excluding regions with missing values. Mean signal intensities were calculated for control and SOX21-activated samples. Log_2 fold changes were computed as $log_2FC = log_2((treat + 1) / (control + 1))$. Statistical significance was assessed using Welch's t-test, and global median shifts in log_2FC were evaluated by the Wilcoxon signed-rank test.

Gene annotation was performed using GREAT (v3.0.0). Motif enrichment was analyzed using HOMER (findMotifsGenome.pl, default settings), with peak sets serving as reciprocal background controls in two separate HOMER runs.

ATAC-seq

ATAC-seq was performed using three replicates per condition, with 50,000 cells per replicate. Tn5-based ATAC experiments were conducted at SciLife Lab (Stockholm, Sweden), with sequencing performed on an Illumina NextSeq2000, generating paired-end 2x50 bp reads. FastQ reads were processed using the nf-core ATAC-seq bioinformatics pipeline (NGI, SciLife Lab, v2.1.2) for mapping, quality control, peak calling, and annotation. Peak differences were analyzed with DiffBind. Footprint analysis on regions less accessible upon SOX21 induction was performed using TOBIAS after Tn5 insertion bias correction. ATACorrect was used for

global bias correction, and BINDetect and ScoreBigwig were used to normalize subsets of SOX21 ChIP-seq peaks.

RNA-seq

Approximately 200,000 cells per sample were harvested 45-48 hours post-DOX treatment or control conditions. RNA was extracted using the RNeasy Prep Kit (Qiagen) following manufacturer guidelines. RNA-seq libraries were generated with the TruSeq RNA Library Prep Kit v2 (Illumina) and sequenced on a NovoSeq6000, producing 2x150 bp reads. FastQ files underwent demultiplexing, quality control, and alignment to hg38 using the NGI SciLife Lab RNA-seq pipeline (v3.14.0). RNA-seq experiments for AP1-inhibitors and c-JUN over-expression were done by Novogene (www.novogene.com) according to their standards. Differential gene expression analysis was conducted with DESeq2, identifying significantly regulated genes with log2 fold change < -1 or >1 and adjusted p-value < 0.05. Volcano plots were generated using the EnhancedVolcano R package. Heatmaps of RNA-seq read counts for selected Gene Ontology (GO) gene sets were created using the R heatmap function. GO term enrichment was visualized with ToppGene.

To investigate transcriptional relationships between glioblastoma subtypes and experimental samples, we applied Uniform Manifold Approximation and Projection (UMAP) for nonlinear dimensionality reduction. UMAP was trained on a reference cohort of glioblastoma tumors from TCGA (n = 539), using a curated gene set[6] , which defines transcriptional signatures for glioblastoma subtypes (MES, NPC, OPC, AC). The same gene set was used to process experimental transcriptomes (JM11, JM12, JM13) for downstream projection.

Gene expression values were Z-score normalized (mean = 0, standard deviation = 1) across genes. Missing values were imputed as zeros prior to normalization to maintain dimensional

consistency. The UMAP model was trained with n_neighbors=25, min_dist=0.001, spread=1.0, and the Manhattan distance metric.

Experimental samples were projected into the pre-trained UMAP space without altering the model, enabling direct comparison with TCGA tumor profiles. Visualization was performed by coloring samples according to the median expression of Neftel-defined subtype gene sets. Replicates of JM11, JM12, and JM13 were labelled to assess their transcriptional proximity to canonical glioblastoma subtypes.

References

1 McLendon R, Friedman A, Bigner D, Meir EGV, Brat DJ, Mastrogianakis GM *et al.* Comprehensive genomic characterization defines human glioblastoma genes and core pathways. *Nature* 2008; **455**: 1061–1068.

2 Goldman MJ, Craft B, Hastie M, Repečka K, McDade F, Kamath A *et al.* Visualizing and interpreting cancer genomics data via the Xena platform. *Nat Biotechnol* 2020; **38**: 675–678.

3 Zakharova G, Efimov V, Raevskiy M, Rumiantsev P, Gudkov A, Belogurova-Ovchinnikova O *et al.* Reclassification of TCGA Diffuse Glioma Profiles Linked to Transcriptomic, Epigenetic, Genomic and Clinical Data, According to the 2021 WHO CNS Tumor Classification. *Int J Mol Sci* 2022; **24**: 157.

4 Zhao Z, Zhang K-N, Wang Q, Li G, Zeng F, Zhang Y *et al.* Chinese Glioma Genome Atlas (CGGA): A Comprehensive Resource with Functional Genomic Data from Chinese Glioma Patients. *Genom, Proteom Bioinform* 2021; **19**: 1–12.

5 Team RC. R: A Language and Environment for Statistical Computing_. R Foundation for Statistical Computing. 2023.https://www.R-project.org/.

6 Neftel C, Laffy J, Filbin MG, Hara T, Shore ME, Rahme GJ *et al.* An Integrative Model of Cellular States, Plasticity, and Genetics for Glioblastoma. *Cell* 2019; **178**: 835-849.e21.
