# Supplementary material for: SOX21 suppresses glioblastoma growth by repressing AP-1 activity
Source: Cell Death Dis. 2026 Jan 31;17(1):191. doi: 10.1038/s41419-026-08442-5 (PMC12876893; doi:10.1038/s41419-026-08442-5)

Figure 2B

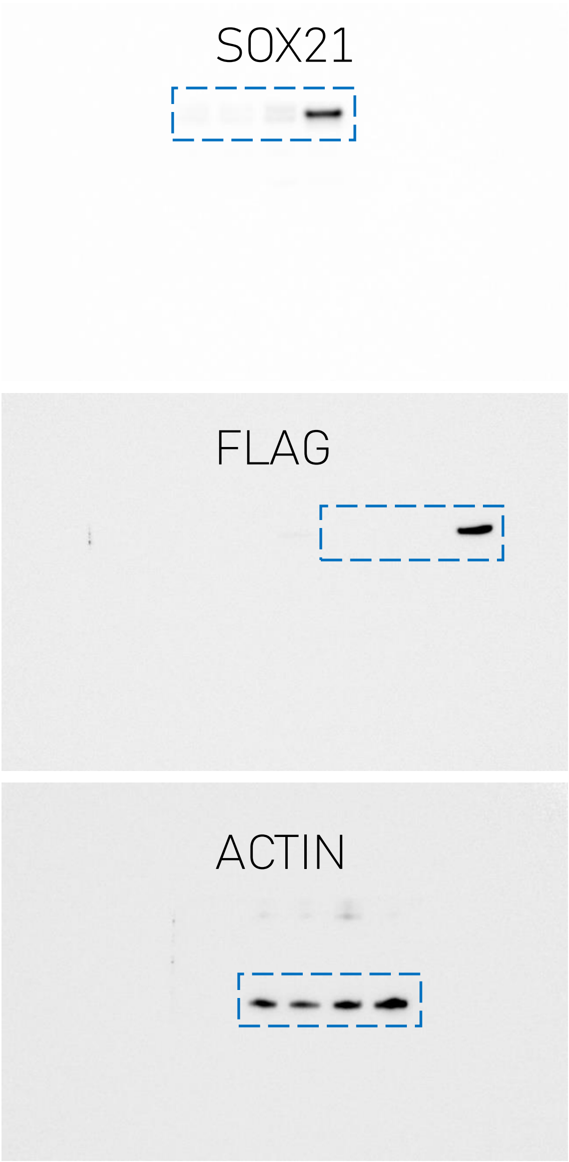

Figure 4H

JUN

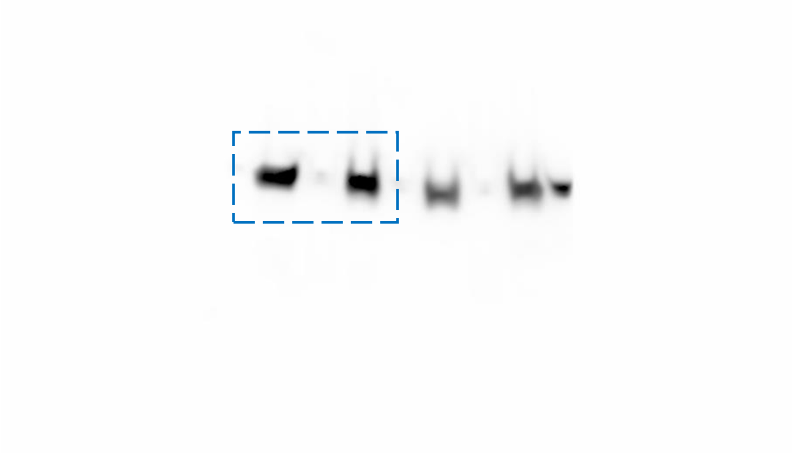

FLAG

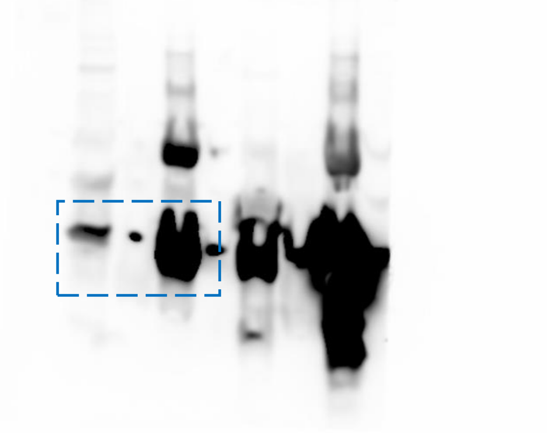

Figure 4I

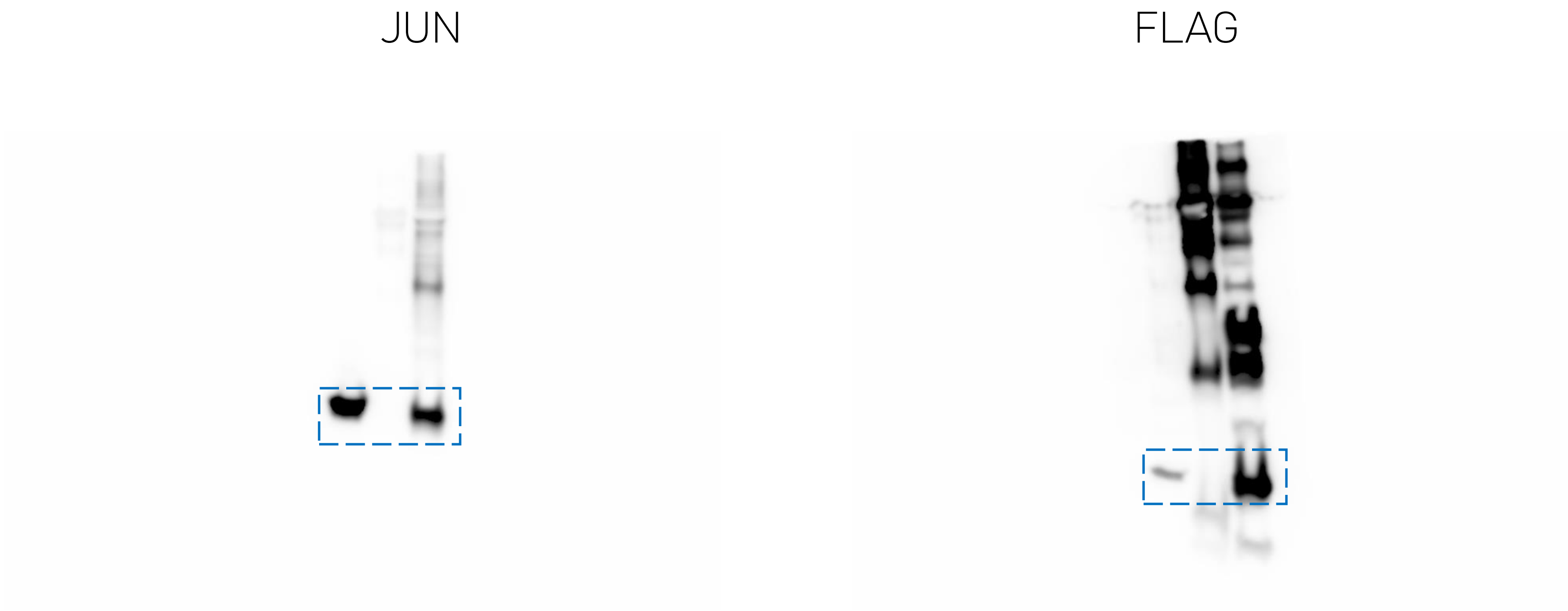

Figure 6E

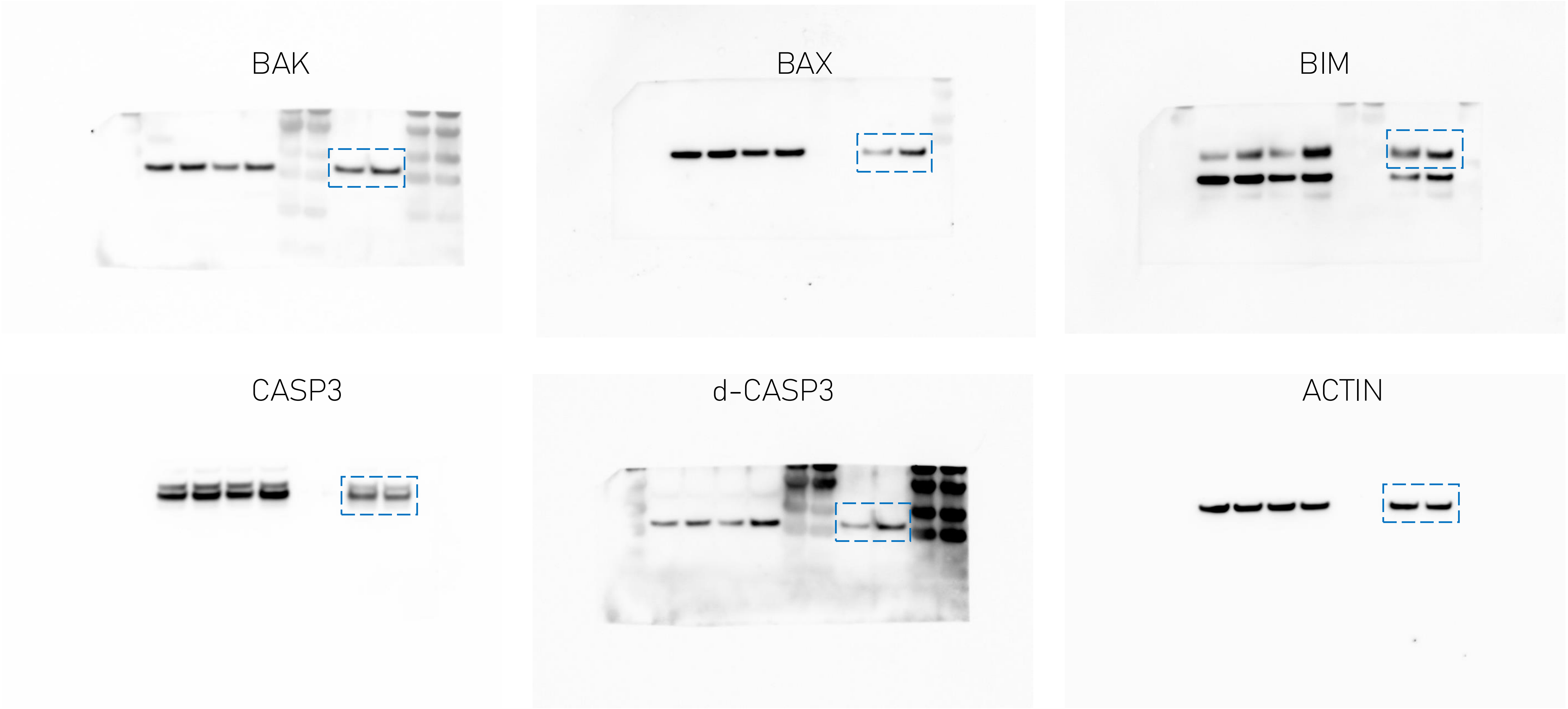

Figure S3A

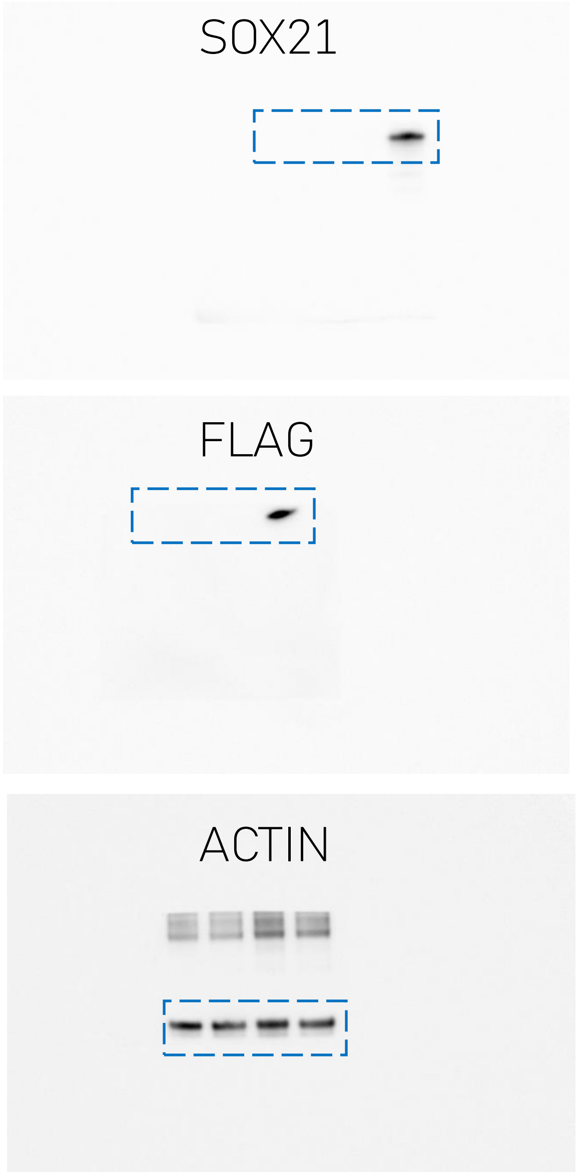

Figure S4C

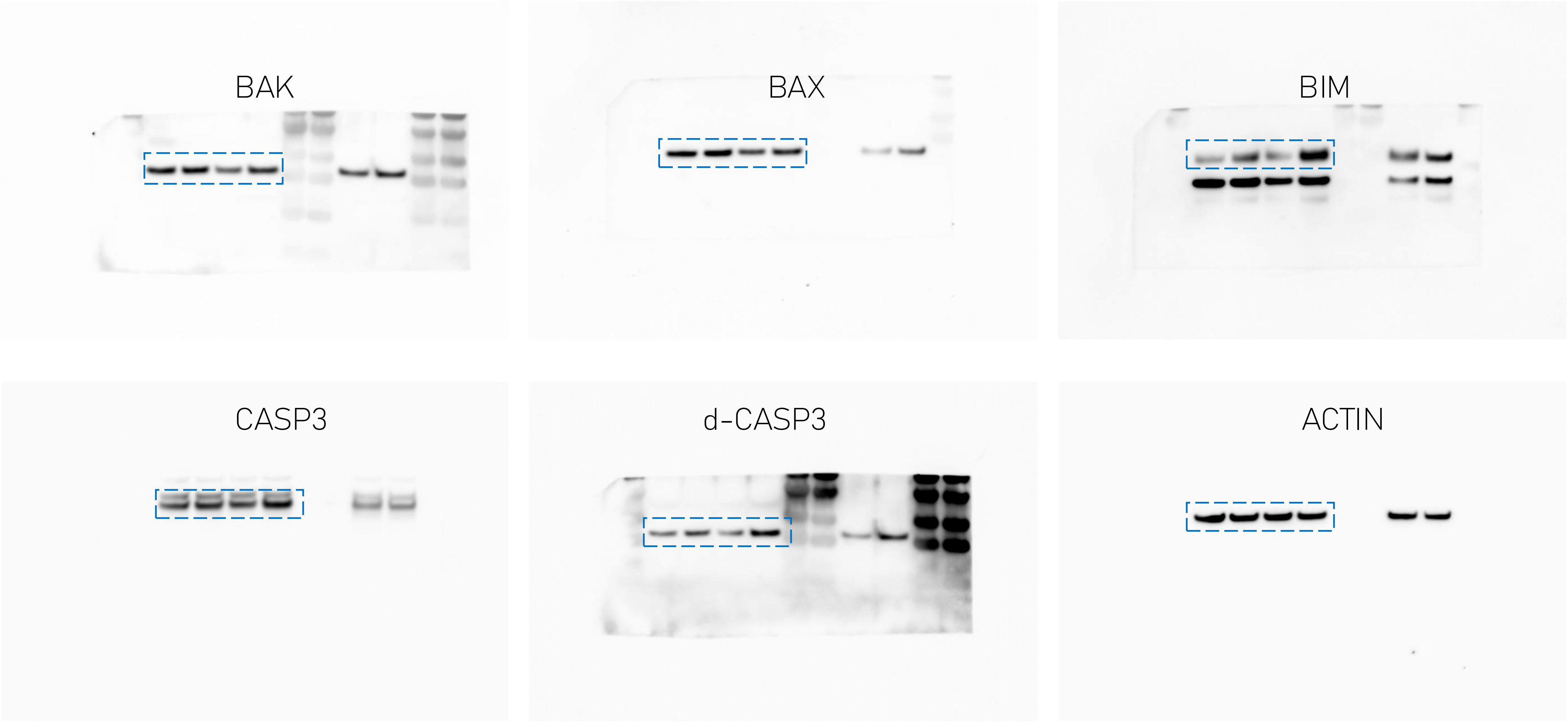

Figure S4D

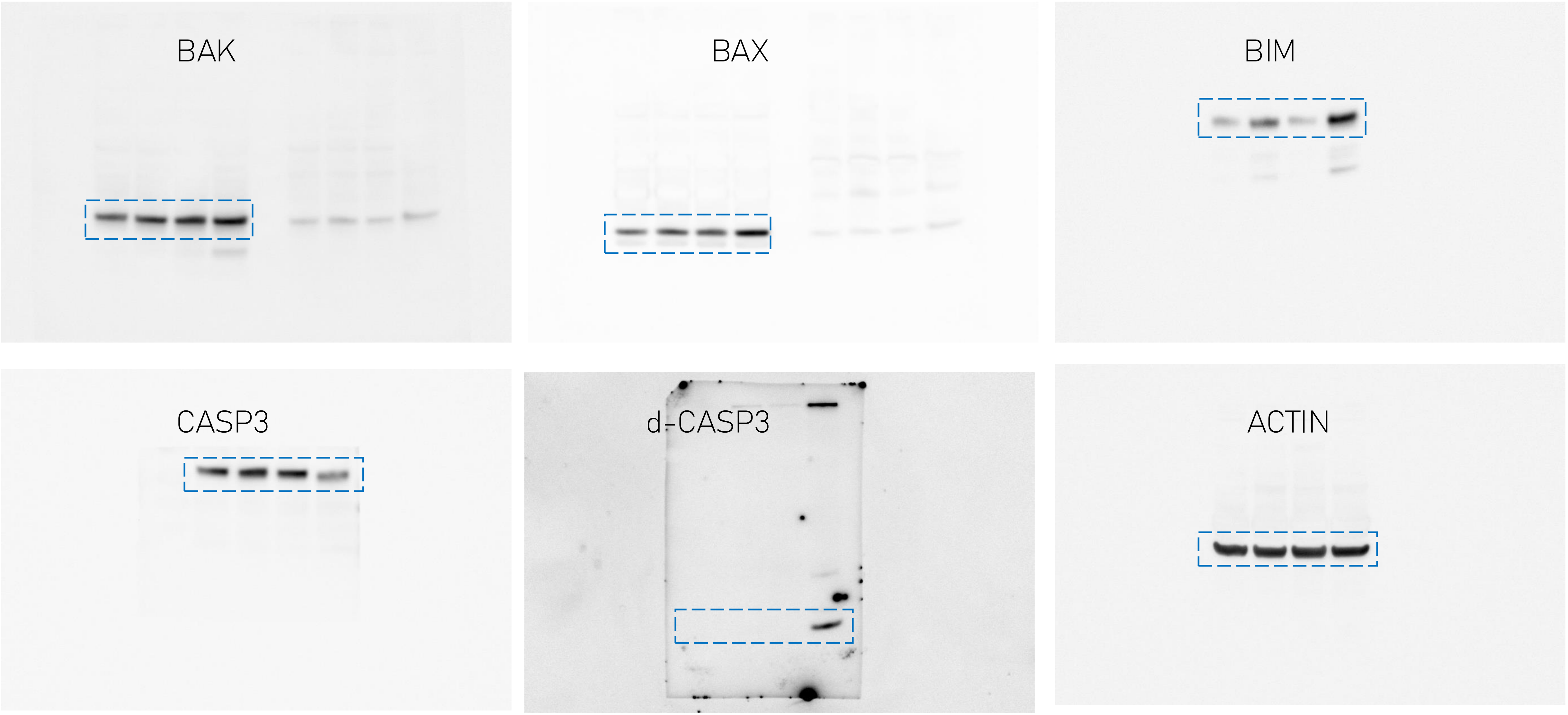

Figure S4E

GPX4

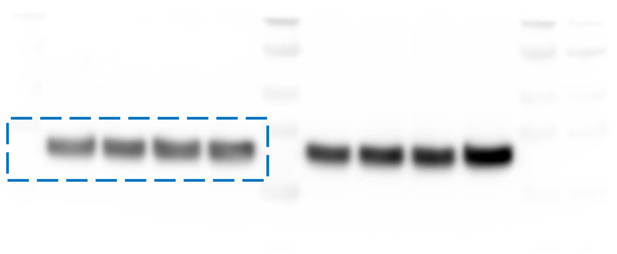

ACTIN

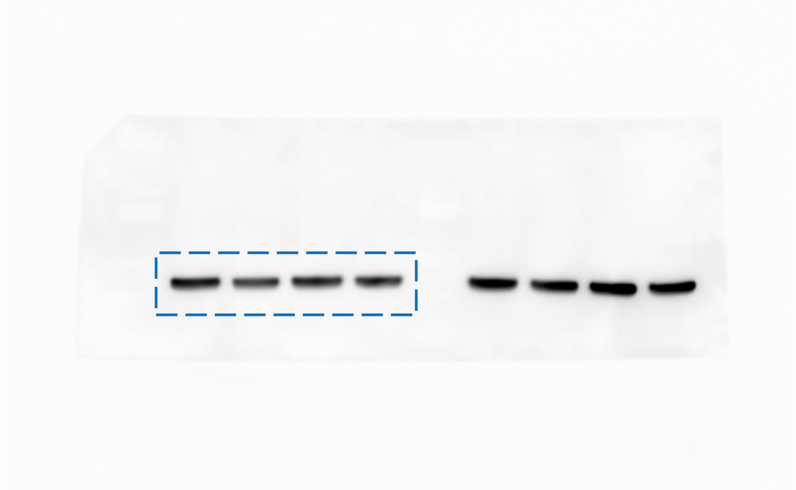

Figure S4F

GPX4

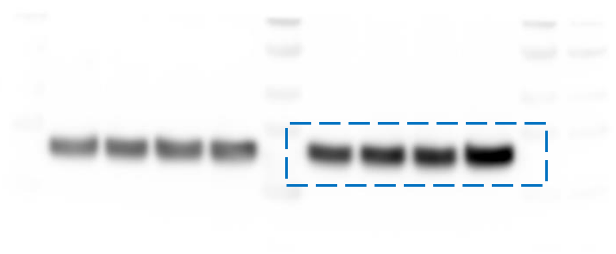

ACTIN

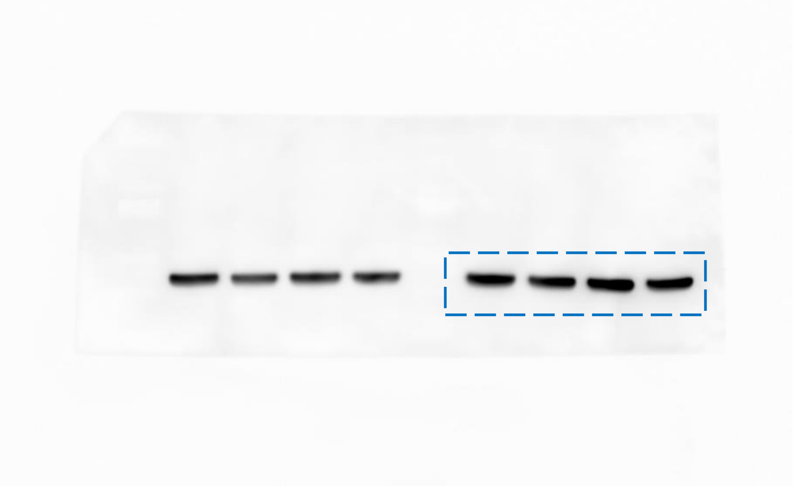

Figure S10D

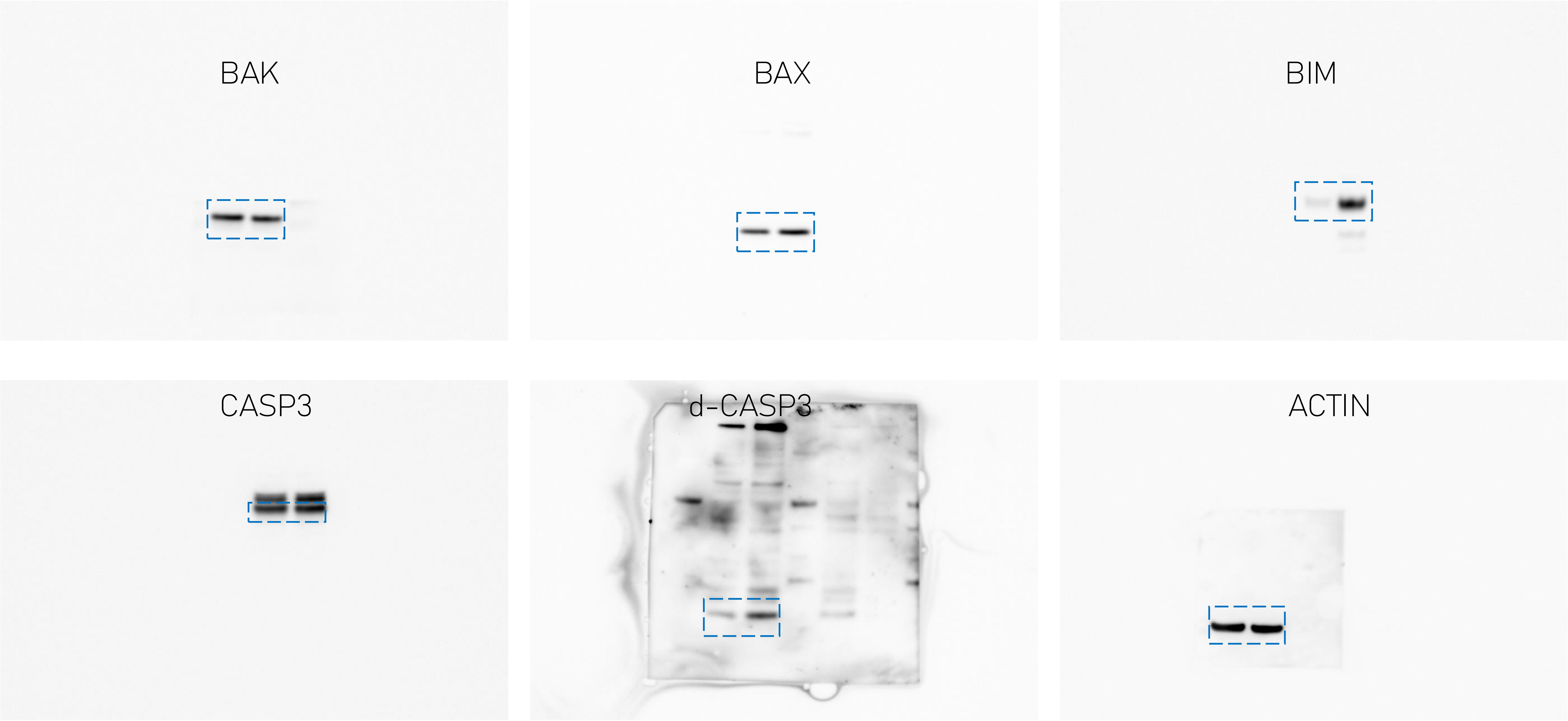

Supplement: Supplementary file 8 — Supplementary WB data [file 41419_2026_8442_MOESM8_ESM.pdf]
